# Comparative Yolk Proteomic Analysis of Fertilized Low and High Cholesterol Eggs during Embryonic Development

**DOI:** 10.3390/ani11030744

**Published:** 2021-03-09

**Authors:** Haji Gul, Xingyong Chen, Zhaoyu Geng

**Affiliations:** 1 College of Animal Science and Technology, Anhui Agricultural University, No. 130 Changjiang West Road, Hefei 230036, China; chenxingyong@ahau.edu.cn; 2 Anhui Province Key Laboratory of Local Livestock and Poultry Genetic Resource Conservation and Bio-Breeding, Anhui Agricultural University, No. 130 Changjiang West Road, Hefei 230036, China

**Keywords:** egg yolk proteins, yolk cholesterol, vitellogenin, Ig-gamma (clone-36 chicken), ovotransferrin, ovoinhibitor, vitellin membrane outer layer protein

## Abstract

**Simple Summary:**

Cholesterol exists in each cell of the living organism and plays a significant role in cellular functions. Several reports have demonstrated that high yolk cholesterol concentration affected embryo mortality, hatchability, and performance. The present results showed that high-cholesterol egg yolks had increased protein intensities and egg and yolk weight relative to low-cholesterol egg yolks. The differentially expressed yolk proteins were primarily involved in lipid transport, lipid localization, nutrient reservoir function, and embryo protection during embryonic development.

**Abstract:**

The yolk is the principal part of the egg that contains vitamins, minerals, lipids, and proteins which are essential for embryo development and hatching. The egg yolk contains significant amounts of lipoproteins, triacylglycerides, and cholesterol, whose dynamics are indistinct during embryogenesis. The effects of cholesterol on the yolk protein abundance, intensity, and function are ill-defined during embryonic development. Using two-dimensional gel electrophoresis, eggs with respective high and low cholesterol protein abundance were investigated after 0, 2, 6, and 13 days of embryogenesis and further analyzed by matrix-assisted laser desorption/ionization time-of-flight tandem mass spectrometry. The results revealed that the vitellogenin proteins are the most abundant egg yolk protein that showed proximity and a high degree of variation in isoelectric point and molecular weight. The results demonstrated increased expression of vitellogenin-1 and vitellogenin-3 at two days and vitellogenin-2 protein at 13 days of embryogenesis in both egg types. The ovoinhibitor, immunoglobulin lambda light chain precursor, Ig-gamma (clone-36 chicken), and beta-2-glycoprotein-1 precursor proteins were significantly expressed in high cholesterol eggs while haptoglobin protein PIT-54 and vitelline membrane outer layer proteins intensities were significant in low cholesterol eggs at two days of embryogenesis. The high cholesterol eggs showed a modest increase in egg weight, yolk weight, albumen height, yolk color, and egg strength relative to the low cholesterol eggs. The gene ontology enrichment analysis revealed that the differentially expressed proteins such as vitellogenin proteins were involved in lipid transport and lipid localization biological processes and showed nutrient reservoir activity function. The ovotransferrin regulated the biological processes of plasminogen activation and extracellular matrix disassembly and characterized the anchored component of the plasma membrane. The ovoinhibitor protein was involved in response to mineralocorticoid and corticosterone biological processes whereas the vitellin membrane outer layer protein constituted the extracellular exosome, extracellular organelle, and membrane-bounded vesicle cellular components. Collectively, our study revealed yolk protein abundance, molecular function, cellular components, and biological processes and concluded that yolk protein intensities were significantly altered by cholesterol concentration.

## 1. Introduction

The hen egg is considered a good food emulsifier and is widely used in baked goods, sauces, and salad dressings due to its gelling, whipping, and emulsification properties. An egg contains a variety of bioactive compounds and comprised of two parts: the egg white and the yolk. The egg white contains essential nutrients, antimicrobial factors, and provides nourishment to developing embryo [1,2,3]. The egg yolk had approximately 50% total dry matter, 17% protein, and 33% lipids [4,5]. The yolk proteins are divided into two parts; the yolk plasma contains livetin and low-density lipoprotein (LDL) and the granules part contains high-density lipoprotein (HDL) and phosvitin [6]. The plasma contains 85% of LDL while granules contain 70% HDL [7]. Egg lipids per 100 g of fresh yolk consist of 13.2 g monounsaturated fatty acids, 8.7 g saturated fatty acids, 3.4 g polyunsaturated fatty acids, and 1.12 mg cholesterol [8]. Previous studies showed that cholesterol intake from eggs can cause hyperlipidemia, dyslipidemia, and atherosclerosis [9,10] whereas other investigations revealed that the yolk cholesterol concentration affected embryo mortality and performance during hatching [11,12]; for example, a high amount of yolk cholesterol decreased the hatchability [13]. The study also revealed that the yolk cholesterol varied among different chicken breed and is regulated by genetic factors [12] such as a decrease in yolk cholesterol increased apolipoprotein H and cholesterol-7 alpha-hydroxylase while decreased vitellogenin and apolipoprotein B expression level [14]. The rate of cholesterol synthesis is high during embryo development [15] and the fetus with a low level of cholesterol synthesis had numerous congenital defects and abnormalities [16,17]. The significance of cholesterol is such that when cholesterol is substituted by its nonfunctional analog i.e., 25-azacoprostane, the embryo suffers growth defects and the development of animal is inhibited [18]. In our previous study, the high-cholesterol egg yolk had significantly higher cohesiveness than the low-cholesterol egg yolk; however, egg white hardness, gelling properties, and water retention ability were unaffected [19]. In addition, the yolk cholesterol concentration decreased the foaming capacity of the yolk in the low cholesterol egg compared to the control [19,20]. In other animals, cholesterol maintains membrane integrity as well as the structure and function of membrane-bound proteins [21,22] and are involved in directing the activity of proteins into lipid membrane microdomains [23]. The egg yolk cholesterol is the precursor for steroid hormones, such as mineralocorticoids and glucocorticoids that are essential for embryo development. For example, a lack of the glucocorticoid leads to neonatal mortality soon after birth due to growth development inhibition [24]. The fundamental role of the yolk proteins and lipids is to fulfill the physiological and nutritional requirements of embryo during embryogenesis [25] that consists of three basic stages, namely, embryo implantations (first week), embryo completion (second week), and embryo hatching (last week). The initial two weeks of embryogenesis are essential whereby the embryo utilized albumen and yolk ingredients, and moves towards the hatching place. In the last week, the embryo utilized the nutrients orally [2,25].

The chick embryo undergoes multiple cleavages during the first week and the cholesterol biosynthesis begins at the periimplantation stage (embryonic day 2–6) while in the second week, the trophoblasts, uterine endometrium, embryoblast, and embryo formation occurred [26,27]. During this phase, maternal blood and remnants of trophoblast cell digestion served as a source of cholesterol for the conceptus [28]. Previous work demonstrated that during embryonic development, certain egg white and yolk proteins such as albumen and ovotransferrin abundance decreased, while the intensities of some proteins increased during the first and second week of embryogenesis [10,29,30,31]. Importantly, the second week of embryogenesis is regarded as the primary stage in the modification of egg yolk protein and our previous work revealed 12 proteins that showed major changes in molecular weight (MW), isoelectric points (pI), and protein expression level [19]. Hence, it is highly intriguing to examine the modification of egg yolk proteins in low and high cholesterol eggs during the early phase of embryogenesis, specifically the first and second weeks of incubation.

In the present study, the effects of low and high cholesterol were studied on the egg yolk protein by comparing the changes at the proteomic level after 0, 2, 6, and 13 days of embryogenesis. The vitellogenin proteins were detected in high abundance in egg yolks with 23 protein spots; among them, 6 were vitellogenin-1, 14 vitellogenin-2, and 3 were vitellogenin-3. Fourteen spots were detected in the immunoglobulin proteins in which 9 spots were Ig-gamma (clone-36 chicken), 3 spots were immunoglobulin lambda light chain precursors, 2 spots were the immunoglobulin Y heavy-chain constant region, and 1 spot was the immunoglobulin light chain precursor protein. In addition, ovoinhibitors 9 spots, ovotransferrin 6 spots, and in serum albumin, 5 spots were detected in both egg types by two-dimensional (2-D) gel-electrophoresis and matrix-assisted laser desorption/ionization time-of-flight tandem mass spectrometry (MALDI-TOF-MS/MS). The majority of proteins showed a high degree of variation in pI and MW. The cholesterol content induced changes in protein concentration that reflects a potential role of cholesterol in changing intensities of egg yolk proteins.

## 2. Materials and Methods

The animal management guidelines of the China Council on Animal Care were followed and the experimental protocols were approved by the animal care committee of Anhui Agricultural University (No. SYDW-P2018110702).

### 2.1. Experimental Design

A total of 1080 Huainan ephedra laying hens at age of 40 weeks, with similar body weights (1.48 ± 0.13 kg) were allocated into four groups. Hens were housed into 30 battery cages (as 30 replicates, 6 tiers, and 6 cages per tier) with one hen per cage. Thirty eggs were collected from each group (one egg per replicate) within three consecutive days for egg quality and yolk cholesterol determination. The egg yolk cholesterol content was determined and the lowest and the highest cholesterol eggs group were selected. All the laying hens were treated uniformly under a light/dark cycle of 16 h light and 8 h darkness (16L:8D) and allowed free access to feed and water. The selected two groups (low and high cholesterol) fertilized eggs were collected from the poultry research center farm of Anhui Agricultural University. Thirty-six eggs were randomly selected on 0, 2, 6, and 13 days of incubation with nine in each time point. Eggs collected at 0 days were fertilized eggs and without incubation. The incubation temperature was 37.8 ± 0.5 °C with a relative humidity of 60%.

### 2.2. Egg Quality Assessment

Eggs quality was analyzed according to the Chen et al. procedure [32]. Egg weight was determined by digital scale weight balance (accuracy: 0.01 g) and egg strength was measured by eggshell force gauge (Robotmation Co., Ltd., Tokyo, Japan). The shell thickness was measured by digital Vernier caliper (NFN380, Fujihira Industry Co., LTD., Tokyo, Japan), specific gravity was measured by weighing the egg divided by the volume of the egg, and the egg shape index was determined from the ratio of longitudinal diameter and the transverse diameter of each egg by an electronic digital caliper (model II, Robotmation Co., Ltd., Tokyo, Japan). An automatic egg analyzer (Egg Multi Tester, EMT-5200, Robotmation Co., Ltd., Tokyo, Japan) was used to measure Haugh units, albumen height, and yolk color.

### 2.3. Yolk Cholesterol Analysis

For egg yolk cholesterol analysis, the Chen et al. procedure was followed [32]. Briefly, 0.1 g of the yolk sample was taken in a 1.5 mL tube with anhydrous ethanol and homogenized mechanically for 30 s at 50 Hz. The samples were centrifuged for 10 min at 2500 rpm and 25 µL of the supernatant was transferred to a 96-well plate. Next, 250 μL of the working solution was added and the mixture was incubated for 10 min. The composition of the working solution was; 50 mmol/L Good’s buffer, 5 mmol/L phenol, 0.3 mmol/L 4-AAP, ≥50 KU/L cholesteryl esterase, ≥25 KU/L cholesterol oxidase, and ≥1.3 KU/L peroxidase. The cholesterol content was measured at wavelength 510 nm by the following formula.

Cholesterol content (mg) = (sample-OD_510_ − blank-OD_510_)/(corrected-OD_510_ − blank-OD_510_) × dilution factor × yolk weight × 386.6535/1000.

### 2.4. Egg Yolk Protein Extraction

Protein extraction from egg yolk was performed according to Mann and Mann, (2008) procedure [5]. Briefly, 0.2 g of egg yolk was added in 1 mL ice-cold lysis buffer (5 μL of phosphatase inhibitor, 1 μL of protease inhibitor, 10 μL of phenylmethanesulfonyl fluoride) and then homogenized at 4 °C for 3 min. The samples were centrifuged at 12,000× *g* for 10 min at 4 °C and whole yolk protein extract was collected. For desalination of yolk protein extract, a 100 mL extracted protein was mix with 400 mL of ice-cold acetone and kept overnight at −20 °C, then the sample was centrifuged at 12,000× *g* for 10 min and the pellet was air-dried for 15 min in a draft cupboard (FGG1500, Kebei, Wuhan, China) and then through rehydration buffer-1 (8-mol urea, 4% CHAPS, 65-mmol dl-dithiothreitol, Bio-Lyte 0.2% (w/v), and bromophenol blue 0.001%) resolubilized. The samples were homogenized for 10 min and then centrifuged at 1000× *g* for 10 min at 4 °C. The supernatant was collected and stored at −20 °C. Bradford protein quantification kit was used for the quantification of protein samples (Yeasen, Shanghai, China).

### 2.5. The 2-D Gel Electrophoresis and Proteins Identification

The 2-D gel electrophoresis was performed by the EttanIPGphor3 system (GE Healthcare, Chicago, IL, USA). The first dimension was isoelectric focusing (IEF) by using the EthanIPHphor3 system and the range of pH was 3–10 while the second dimension was Ettan DALT Six System (GE Healthcare, USA) for SDS-PAGE. The experiments were performed in triplicates and the 2-D PAGE Naveena et al. protocol was followed [33]. Proteins spots with good reproducibility were selected for mass spectrometry analysis, excised manually from the gel, destined, washed, and digested with trypsin (Promega, USA). The samples were alkylated, mixed with an equivalent matrix solution (HCCA), and MALDI-TOF-MS/MS analysis was performed using a fuzzy logic feedback control system (Ultraflex MALDI-TOF-TOF mass spectrometer Bruker Karlsruhe, Germany) [29]. The identification of proteins was performed by searching the MASCOT program (http://www.matrixscience.com, accessed on 29 December 2020) in the non-redundant sequence database of NCBI, and those with identification score <30 were excluded.

### 2.6. Statistical Analysis

GraphPad Prism 8 and IBM SPSS Statistics 25 were used for statistical analysis. One-way analysis of variance and Tukey multiple comparison test determined the significant differences in the group and between the groups. The P-value less than 0.05 was considered as significant difference and asterisks (* *p* < 0.05, ** *p* < 0.005, *** *p* < 0.0001) indicate the lower and higher P-values that simply reflects the degree of data compatibility with the test hypothesis [34,35]. The gels spots were analyzed by PDQuest software, Bio-Rad, and the phylogenetic tree was constructed by Phylogeny.fr (accessed on 4 November 2020, from http://www.phylogeny.fr). For gene ontology (GO) analysis, the proteomics data were analyzed by OmicsBean (accessed on 27 November 2020, from http://www.omicsbean.com:88/) and each protein was distributed in biological function, cellular component, and molecular function. The protein spots at 0 days of embryonic development were considered as a control for two days, two days of incubation was set as a control for six days, and six days of incubation was set as a control for 13 days of embryonic development. The data are from three independent replicates with values ± standard deviation (SD).

## 3. Results

### 3.1. Differential Expression of Yolk Protein in Low and High Cholesterol Eggs

**Figure 1:**
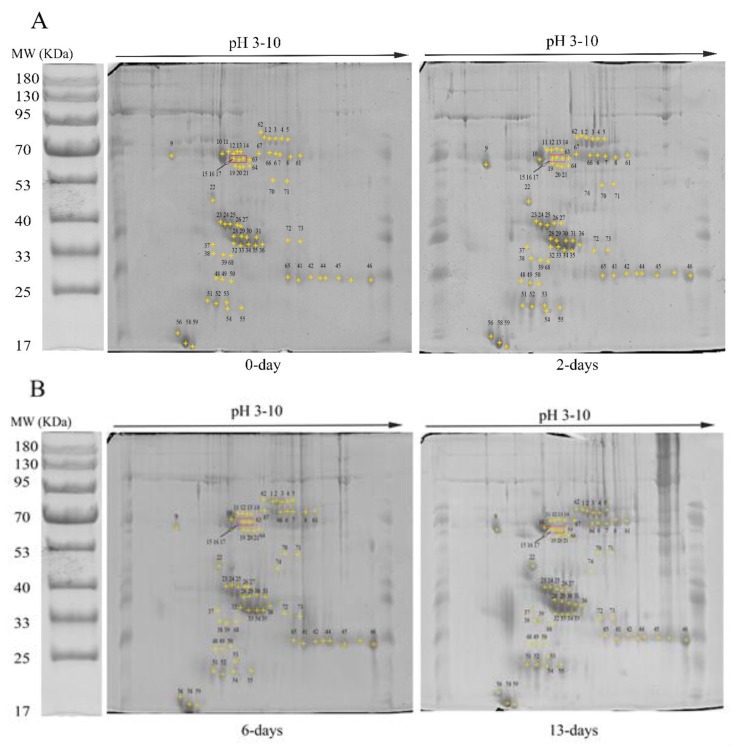

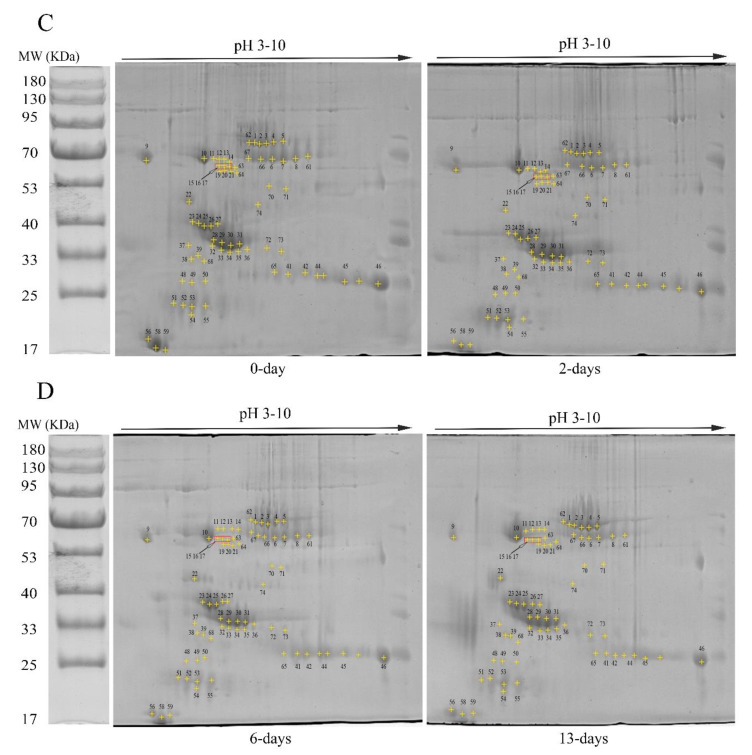
(**A**–**D**) Representative 2-D gel images of the low (**A**,**B**) and high cholesterol (**C**,**D**) eggs proteins after 0, 2, 6, and 13 days of embryonic development. The protein spots were separated by IEF/SDS-PAGE and gels were silver stained. The pI scales, incubation days, and MW are shown. Each gel is representative of three independent replicates.

The results determined the egg yolk proteins abundance in low and high cholesterol eggs by 2-D gel electrophoresis and detected 74 spots in both gels representing 61 and 52 proteins in low and high-cholesterol egg yolks, respectively (Figure 1A–D). The gel at 0 days was used as a control for two days of incubation, the two-days gel was used as a control for six days of incubation, and the six days gel was set as control for 13 days of incubation and the results were analyzed likewise (Figure 1A–D).

**Figure 2:**
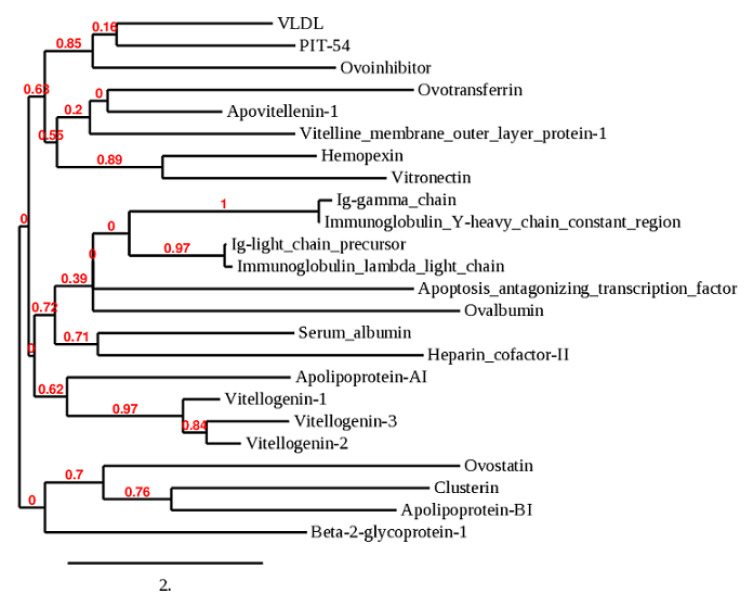
The phylogenetic tree was constructed to check the proximity of detected egg yolk proteins. The vitellogenin proteins (VTG-1, VTG-2, and VTG-3) showed close resemblance and attached in a branch with apolipoprotein-1. Ovotransferrin is associated with apovitellenin-1, serum albumin formed a clade with heparin cofactor-II, ovoinhibitor showed proximity with PIT-54, and vitelline membrane outer layer protein-1 connected with ovotransferrin. The nodes and branch lengths are indicated.

The yolk cholesterol concentration in low cholesterol and high cholesterol eggs were 30.07 ± 1.147 and 40.27 ± 1.022 mg/g/egg, respectively. We collected yolk samples from low and high cholesterol fertilized eggs at day 0, 2, 6, and 13 days of incubation. Samples collected on day 0 were treated as control (fertilized eggs without incubation) while samples collected on days 2, 6, and 13 showed different incubation periods. We detected three types of vitellogenin proteins i.e., spots 23–27 and 50 were identified as vitellogenin-I (VTG-1), spots 28–36 and 43–46, and 65 were identified as vitellogenin-2 (VTG-2), spots 48, 49, and 74 were identified as vitellogenin-3 (VTG-3) protein in low- and high-cholesterol eggs (Figure 1A–D). The vitellogenin proteins showed a close resemblance with each other, such as VTG-1 showed 37% sequence similarity to VTG-2 and 35% to VTG-3 and formed a single clade in the phylogenetic tree as shown in Figure 2.

**Figure 3:**
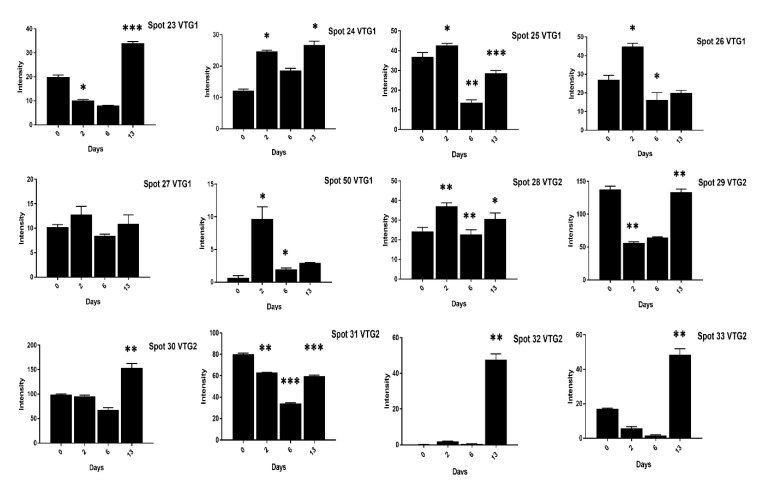
The changes in the intensity of fertilized egg yolk proteins corresponding to Figure 1 has been shown. The VTG-1 and VTG-2 protein spots expression at 0, 2, 6, and 13 days of incubation are indicated in high cholesterol eggs. The column profiles of VTG-1 (23–27, 50) and VTG-2 (28–33) are shown as bar charts with significant differences. The significant differences were determined from the comparison with the control and the asterisks denote statistically significant differences. The data represent the mean values (±SD) from three replicates. * *p* < 0.05, ** *p* < 0.005, *** *p* < 0.0001.

We found vitellogenin proteins as the most abundant egg yolk protein and their expression varied at different days of incubation. For example, at two days of incubation the VTG-1 spots (24–26, 50) expression was significant in high cholesterol eggs (Figure 3) while spots (24–27, 50) expression was high in low cholesterol eggs.

**Figure 4:**
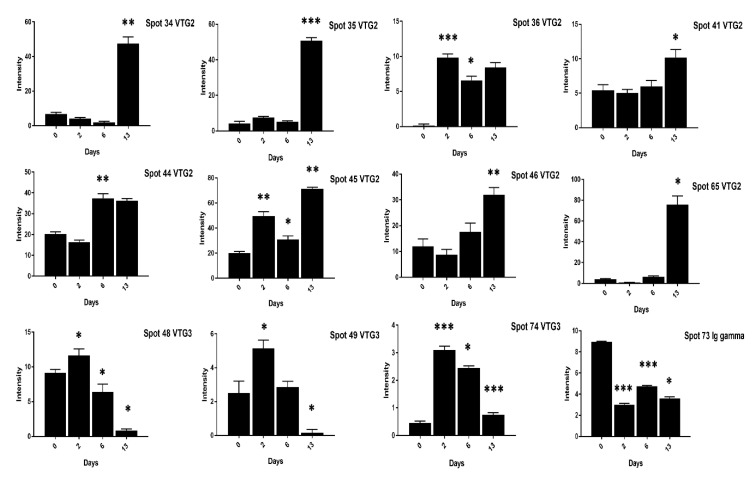
The changes in the intensity of egg yolk proteins at 0, 2, 6, and 13 days of incubation are shown in high cholesterol eggs. The column profiles of VTG-2 spots (34–36, 41, 44–46, 65) and VTG-3 spots (48, 49, 74) Ig gamma (73) are shown as bar charts with significant differences. The significant differences were determined from the comparison with the control and the asterisks denote statistically significant differences. The data represent the mean values (±SD) from three replicates. * *p* < 0.05, ** *p* < 0.005, *** *p* < 0.0001.

**Figure 5:**
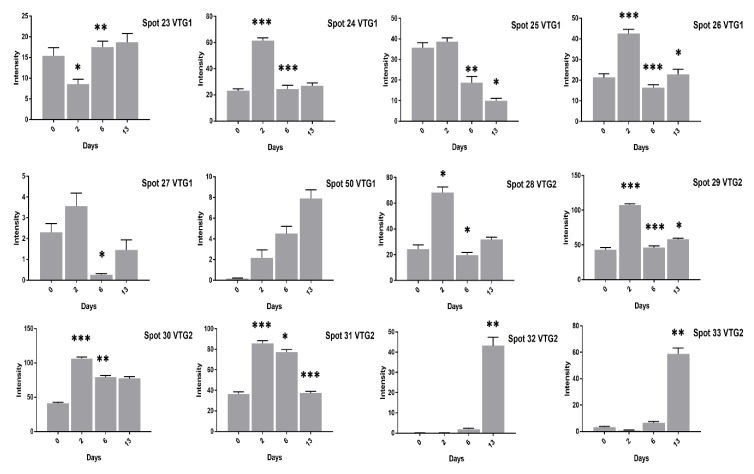
The VTG1 and VTG2 proteins spots expression at 0, 2, 6, and 13 days of incubation are shown in low cholesterol eggs. The column profiles of VTG1 (23–27, 50) and VTG2 (28–33) are shown as bar charts with significant differences. The error bars represent the standard deviation. * *p* < 0.05, ** *p* < 0.005, *** *p* < 0.0001.

**Figure 6:**
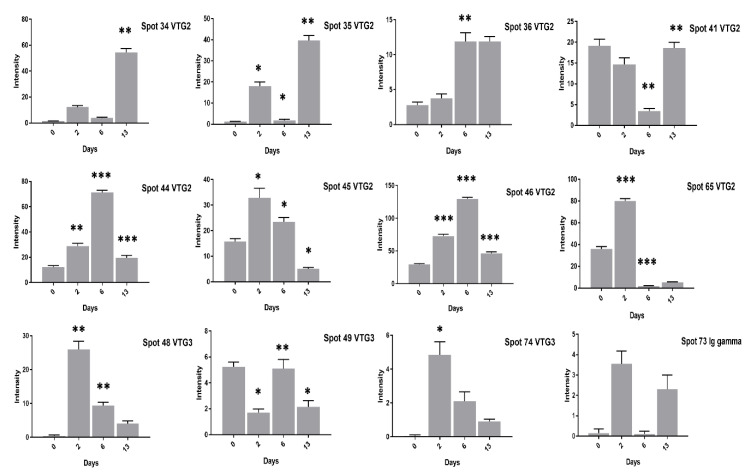
The VTG2, VTG3, and Ig gamma protein spots expression at 0, 2, 6, and 13 days of incubation are shown in low cholesterol eggs. The column profiles of VTG2 spots (34–36, 41, 44–46, 65) and VTG3 spots (48, 49, 74) Ig gamma (73) are indicated as bar charts with significant differences. The error bars represent the standard deviation. * *p* < 0.05, ** *p* < 0.005, *** *p* < 0.0001.

At 13 days of incubation, the VTG-2 protein 12 spots (28–35, 41, 45, 46, 65) in high cholesterol (Figure 3 and Figure 4) and 6 spots (29, 32–35, 41) in low cholesterol eggs (Figure 5 and Figure 6) were highly significant. The VTG-1 protein also showed increased expression after 13 days of incubation in both egg types (Figure 3 and Figure 5). The VTG-3 protein spots (48, 49, 74) showed a consistent decrease in expression with the increase of incubation days in high cholesterol eggs such as at two days of incubation the spots (48, 49, 74) were highly significant while decrease at six days and 13 days of embryogenesis (Figure 4).

Some VTG-2 protein spots (28–31, 34–36, 44–46, 65) showed high expression at two days of incubation in low cholesterol eggs only (Figure 5 and Figure 6).

**Figure 7:**
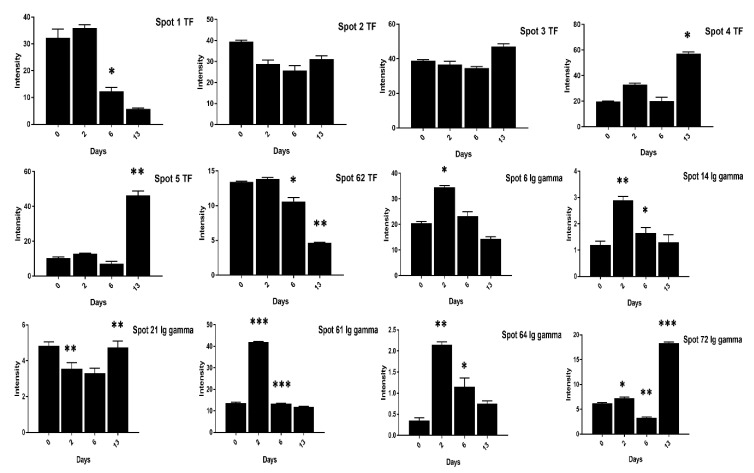
Differential expression of TF and Ig gamma proteins after 0, 2, 6, and 13 days of incubation was shown in high cholesterol eggs. The TF spots (1–5, 62), and Ig gamma (6, 14, 21, 61, 64, 72) showed significant differences at different incubation days. The error bars represent the standard deviation. * *p* < 0.05, ** *p* < 0.005, *** *p* < 0.0001.

Similarly, the VTG-3 protein spots (48, 74) expression was significant at two days of incubation while decreased at 13 days of incubation in low cholesterol eggs (Figure 6). The ovotransferrin (TF) protein is widely reported as egg white protein and six spots (1–5, 62) were detected that showed a consistent decrease from two days to six days and a marked increase at 13 days of incubation in high cholesterol eggs (Figure 7).

**Figure 8:**
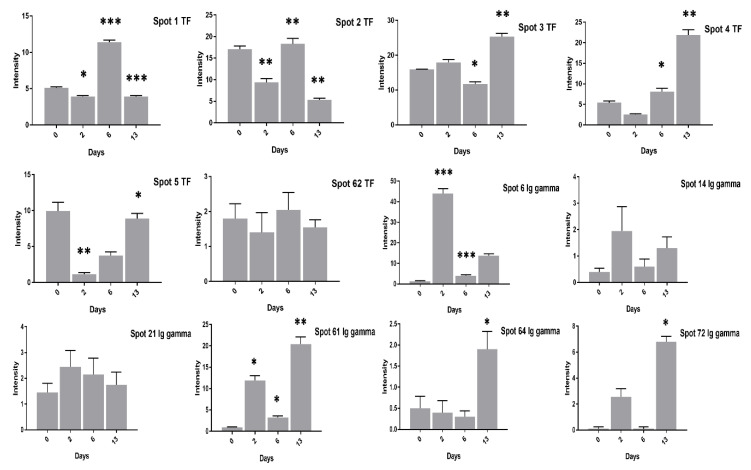
Changes in the intensity of TF and Ig gamma proteins after 0, 2, 6, and 13 days of incubation was shown in low cholesterol eggs. Column profiles of TF (1–5, 63) and Ig gamma (6, 14, 21, 61, 64, 72) protein spots were shown as bar charts. Asterisks denote significant differences at different incubation days. The error bars represent the standard deviation. * *p* < 0.05, ** *p* < 0.005, *** *p* < 0.0001.

**Figure 9:**
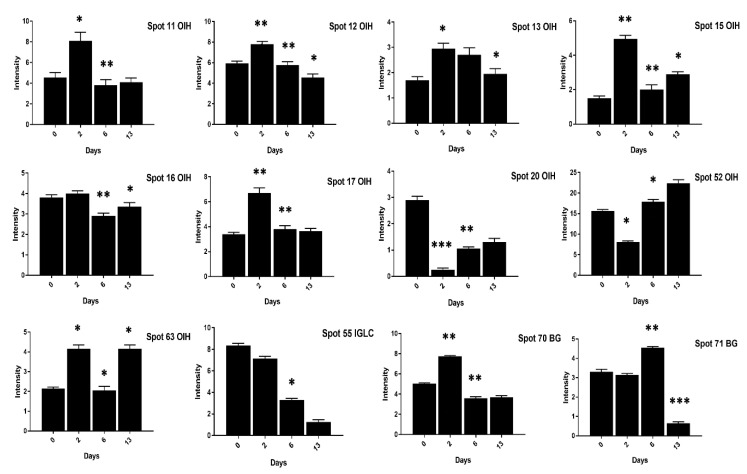
Differential expression of OIH, IGLC, and BG proteins after 0, 2, 6, and 13 days of incubation was shown in high cholesterol eggs. The column profiles of OIH (11–13, 15–17, 20, 52, 63), IGLC (55), and BG (70, 71) are shown as bar charts with significant differences. The values are from three independent replicates and the error bars represent the standard deviation. * *p* < 0.05, ** *p* < 0.005, *** *p* < 0.0001.

**Figure 10:**
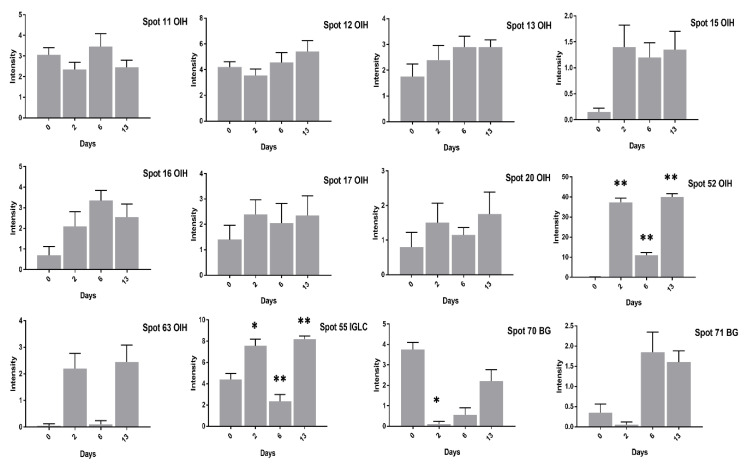
Changes in the intensity of OIH, IGLC and BG proteins spots after 0, 2, 6, and 13 days of incubation was shown in low cholesterol eggs. The column profiles of these proteins are indicated as bar charts with significant differences. The values are from three independent replicates and the error bars represent the standard deviation. * *p* < 0.05, ** *p* < 0.005.

Contrary to the high cholesterol eggs, the TF spots (1, 2, 4, 5, 62) in low cholesterol eggs showed lower expression at two days of incubation and an increase in expression at six days of incubation (Figure 8). The Ig-gamma protein spots (6, 14, 61, 64, 72) intensities were significantly increased in high cholesterol eggs at two days of incubation (Figure 7) while the same spots showed high intensity at two and 13 days of incubation in low cholesterol eggs (Figure 8). At two days of incubation, the ovoinhibitor (OIH) spots (11–13, 15, 17, 63) showed a significant increase while at six days the OIH spots (11–13, 15–17, 20, 52, 63) showed a decrease in high cholesterol eggs (Figure 9). The low cholesterol eggs did not show a significant increase in OIH protein but some spots (13, 15–17, 20, 52, 63) showed a slight increase at two days and a few spots (11–13, 16) at six days of incubation (Figure 10). At 13 days of incubation, the OIH spots showed variation in intensities in both egg types. The immunoglobulin light chain precursor (IGLC) spot (55) expression consistently decrease with incubation days while and beta-2-glycoprotein-1 precursor (BG) spots (70, 71) showed significant increase at two and six days of incubation in high cholesterol eggs (Figure 9). At two days of incubation, the IGLC spot (55) showed significant expression while BG spots (70, 71) showed a decrease in expression in low cholesterol eggs (Figure 10). The spots (37–39) were detected as serum albumin (SA) protein and their abundance was very low in both groups at two days of incubation while their intensities increased at 13 days of incubation in both egg types (Supplementary Materials Figures S1 and S2).

The SA spots (10, 37–39) showed a significant increase at 13 days of incubation in high cholesterol eggs (Supplementary Figure S1) while slight increase in low cholesterol eggs (Supplementary Figure S2). The immunoglobulin lambda light chain precursor (IGLL) spots (51, 53, 54) showed significant changes at two days of incubation while a decrease in intensity at six days of incubation in both egg types (Supplementary Figures S1 and S2). The vitellin membrane outer layer (VMO) protein spot (56) was significant at 6 and 13 days of incubation in high cholesterol eggs (Supplementary Figure S1) while showed significant changes at two and six days of incubation in low cholesterol eggs (Supplementary Figure S2). Some protein spots, such as chicken haptoglobin protein (PIT-54) spot (9), VMO spots (57–59), and immunoglobulin Y-heavy chain constant region (P01875) spots (7, 8), showed variation in expression in both groups (Supplementary Figures S1 and S2).

We also analyzed the low and high cholesterol egg yolk proteins differences in theoretical and experimental isoelectric point and molecular weight (pI/MW) values as shown in Table 1. For example, the theoretical values of Ig gamma protein spots pI/MW were 6.84/54.5 KDa while the experimental pI ranges from 6 to 7.8 and MW 35–70 KDa (Table 1). The Ig gamma spots 72 and 73 showed a lower MW and higher pI values than the theoretical standards. The OIH spots showed a slight variation between theoretical and experimental values. The OIH theoretical pI and MW values were 6.16 and 54.4 KDa and the experimental pI and MW were 5.5–6.0 and 62–68 KDa, respectively.

The OIH spot 52 showed the lowest MW i.e., 23 KDa with 5.5 pI value. The vitellogenin protein spots showed a high variation in pI/MW values. The VTG-1 spots 23–27 and 50 showed a lower experimental MW and pI than the theoretical values. The VTG-1 spots theoretical pI and MW values are 9.16 and 212.6 KDa, respectively while the experimental pI was 5.2–5.8 and MW of all spots was 40 KDa. The VTG-2 spots theoretical pI/MW values were 9.23/206.7 KDa while the experimental pI ranges from 5.5–6.2 and MW ranges 26–36 KDa. The VTG-2 spot-65 showed variation in pI/MW from other spots and its pI was 7.2 and MW 28 KDa. The VTG-3 spots 48, 49, and 74 experimental pI values were 5.4, 5.6, and 6.8 while MW was 28, 28, and 45 KDa respectively. The VTG-3 theoretical pI/MW values were 8.93/193.3 KDa that is higher than the experimental (Table 1). The VMO theoretical pI/MW values were 5.21/21.5 KDa while the experimental pI ranges 3.8–4.0 and MW was 20 KDa. Six TF spots were identified with experimental pI ranges from 7.1–7.8 and MW was 75–77 KDa while theoretical pI/MW values were 7.08/79.6 KDa. Overall, a high degree of variation in theoretical and experimental pI/MW values was detected in vitellogenin proteins, Ig gamma, serum albumin, P01875, PIT-54, BG, and OIH while slight variations were noticed in TF, VMO, and IGLL proteins (Table 1).

### 3.2. Comparison between Low and High Cholesterol Eggs Yolk Proteins

**Figure 11:**
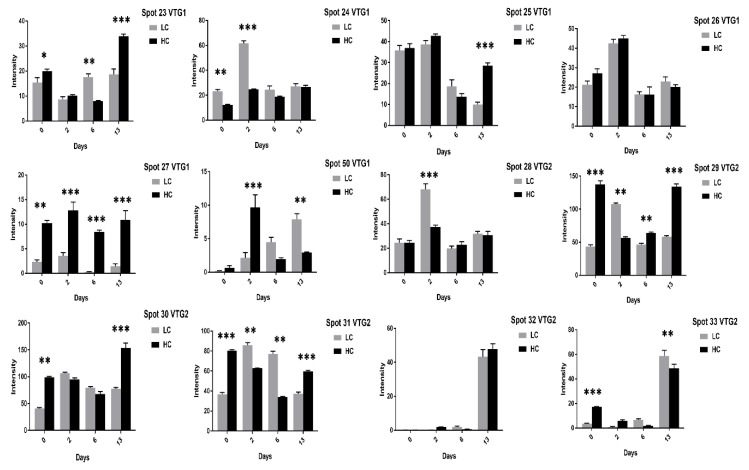
A comparison of low and high cholesterol egg yolk proteins (VTG-1 and VT-2) spots after 0, 2, 6, and 13 days of incubation was performed. The column profiles of VTG-1 and VTG-2 are shown as bar charts with significant differences. The data represent the mean values of three replicates and the error bars denote the standard deviation. * *p* < 0.05, ** *p* < 0.005, *** *p* < 0.0001.

**Figure 12:**
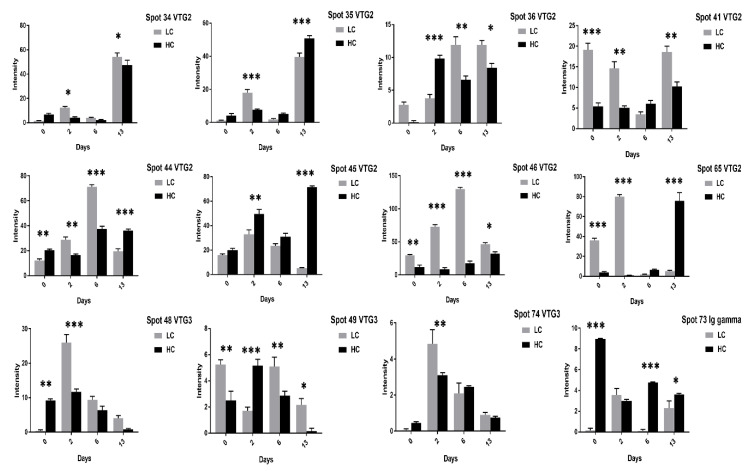
A comparison of low and high cholesterol egg yolk proteins (VTG-2, VTG-3, and Ig gamma) spots after 0, 2, 6, and 13 days of incubation was performed. The column profiles of VTG-2, VTG-3, and Ig gamma are shown as bar charts with significant differences. The data represent the mean values of three replicates and the error bars denote the standard deviation. * *p* < 0.05, ** *p* < 0.005, *** *p* < 0.0001.

**Figure 13:**
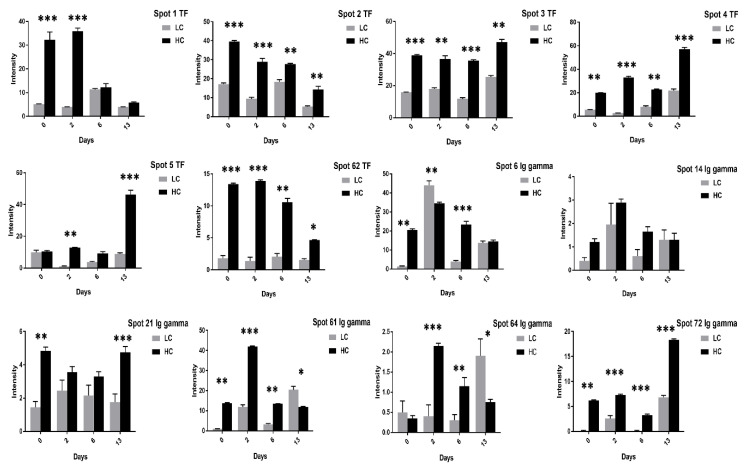
The TF and Ig gamma proteins spots comparative analysis was performed between low and high cholesterol eggs. The TF and Ig gamma spots showed significant differences at different incubation days. The data represent the mean values of three replicates and the error bars denote the standard deviation. * *p* < 0.05, ** *p* < 0.005, *** *p* < 0.0001.

The low and high cholesterol egg yolk proteins were comparatively analyzed at 0, 2, 6, and 13 days of embryogenesis; for example, low cholesterol egg proteins at two days were compared with the corresponding proteins in high cholesterol eggs at two days of incubation. This comparative analysis revealed that the VTG-1 spots (23, 25, 27, 50) and spots (24, 27, 50) were highly significant at 13 and two days of incubation respectively (Figure 11). The VTG-2 protein showed rich enrichment in both egg types but showed variation in the expression at different incubation days such as at 13 days of incubation the VTG-2 spots (29–31, 35, 44, 45, 65) showed a significant increase in high cholesterol eggs (Figure 7) while at two days of incubation the VTG-2 spots (28, 29, 31, 34, 35, 41, 46, 65) were significantly increase in low cholesterol eggs (Figure 11 and Figure 12). The VTG-3 protein spots (48, 49, 74) expression was high in low cholesterol compared to high cholesterol eggs (Figure 12) while TF protein spots (1–5, 62) were significant in high cholesterol compared to low cholesterol eggs (Figure 13).

**Figure 14:**
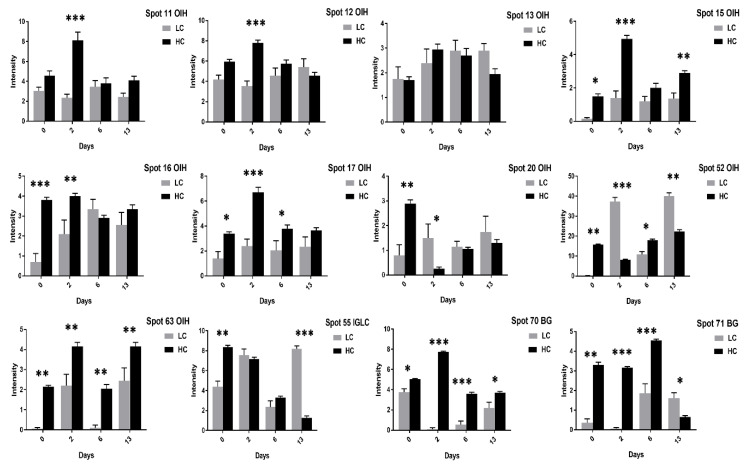
Comparison of OIH, IGLC and BG proteins spots after 0, 2, 6, and 13 days of incubation was shown in high and low cholesterol eggs. The column profiles of OIH (11–13, 15–17, 20, 52, 63), IGLC (55) and BG (70, 71) are shown as bar charts with significant differences. The values are from three independent replicates and the error bars represent the standard deviation. * *p* < 0.05, ** *p* < 0.005, *** *p* < 0.0001.

Furthermore, this comparative study revealed that the Ig gamma protein spots (6, 21, 61, 64, 73) were significantly expressed in high cholesterol eggs while a few spots, such as 61 and 64, were significant in low cholesterol relative to high cholesterol eggs (Figure 13). The majority of OIH protein spots (11, 12, 15–17, 63) and BG spots (70, 71) showed significant expression in high cholesterol compared to low cholesterol eggs (Figure 14).

**Figure 15:**
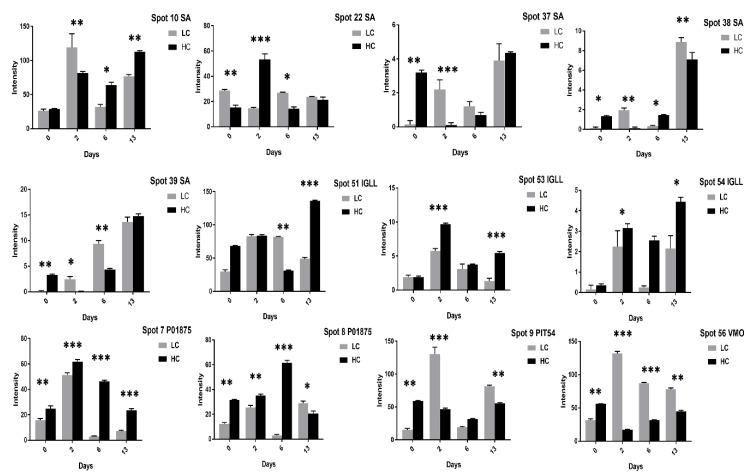
The SA, IGLL, P01875, PIT54, and VMO1 protein intensities were compared in high and low cholesterol eggs at 0, 2, 6, and 13 days of incubation. The column profiles of SA (10, 22, 37–39), IGLL (51, 53, 54), P01875 (7, 8), PIT54 (9), and VMO (56) are shown as bar charts with significant differences. The values are from three independent replicates and the error bars represent the standard deviation. * *p* < 0.05, ** *p* < 0.005, *** *p* < 0.0001.

The SA spots (10, 22, 37–39) comparison showed minor changes in expression in both egg types while the PIT-54 spot (9) and VMO spot (56) expressions were significantly high in low cholesterol eggs (Figure 15). The IGLL and P01875 protein intensities were significant in high cholesterol than low cholesterol eggs (Figure 15). In summary, the comparative study of low and high cholesterol eggs showed that the TF, Ig gamma, OIH, IGLL, P01875, and BG proteins expression were significantly high in high cholesterol relative to low cholesterol eggs (Figure 13, Figure 14 and Figure 15) while PIT-54, VTG-3, and VMO proteins expression were high in low cholesterol than high cholesterol eggs (Figure 12 and Figure 15). Moreover, this comparison also showed that the high cholesterol eggs showed a distinct increase in egg weight, yolk weight, albumin height, yolk color, and egg strength while the egg’s shape, thickness, specific gravity, and Haugh unit values were similar in both egg types (Table 2).

### 3.3. Gene Ontology (GO) Enrichment Analysis of Differentially Expressed Proteins in Biological Process

**Figure 16:**
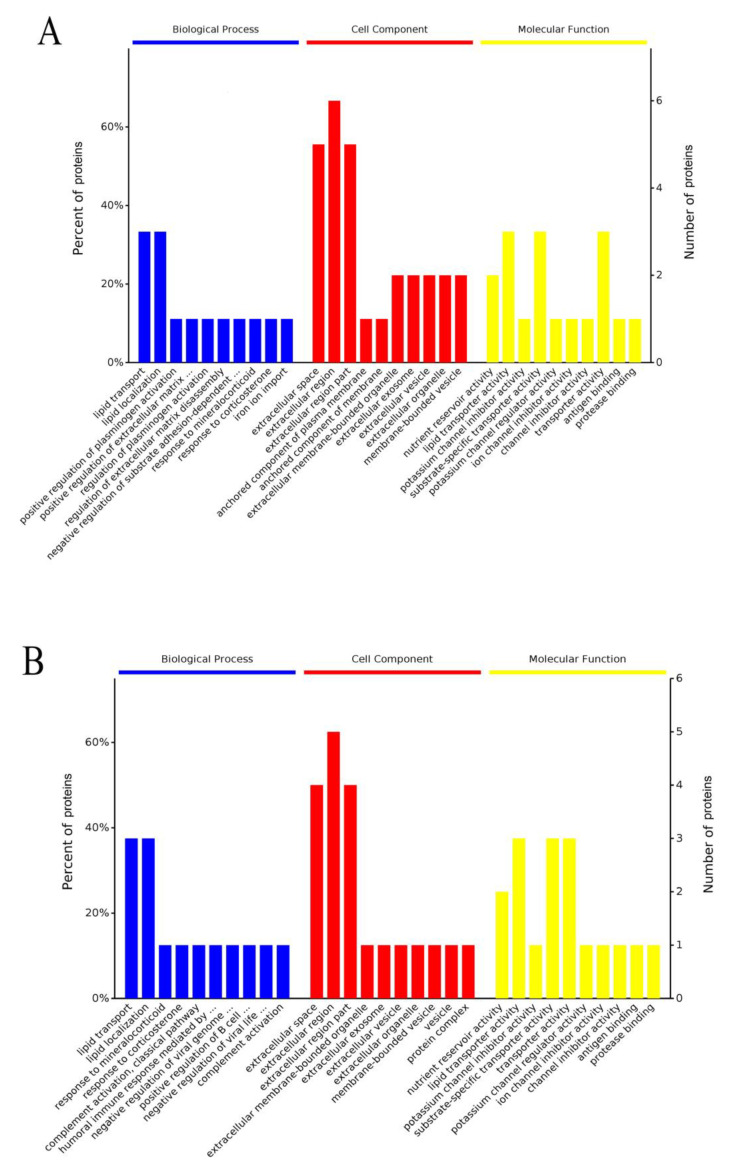

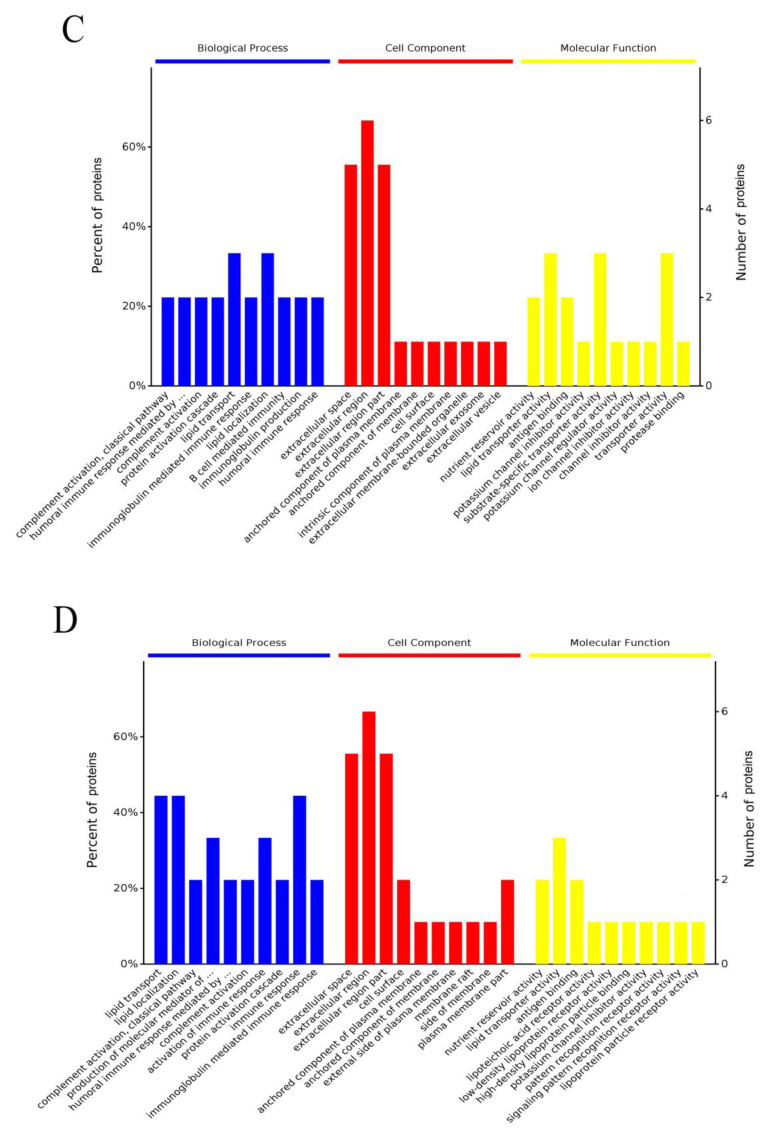

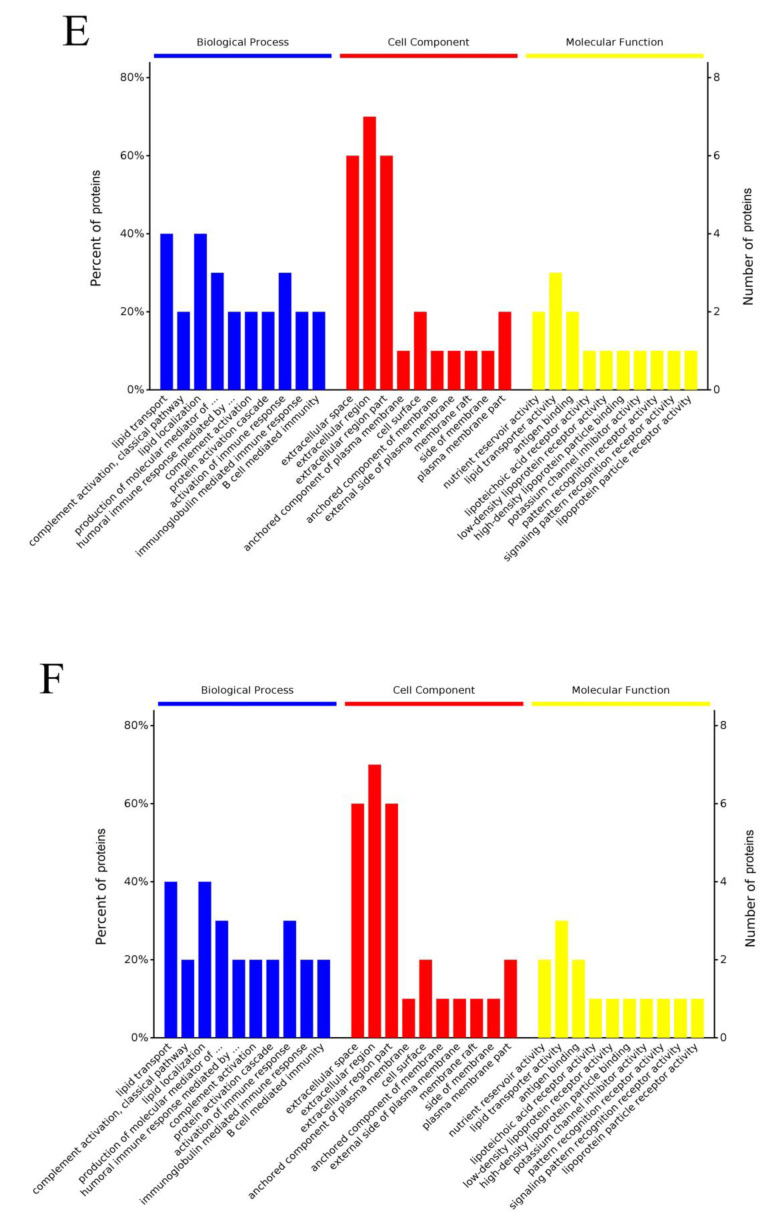
(**A**–**F**) The GO annotation of differentially expressed proteins after two days (**A**,**B**), six days (**C**,**D**), and after 13 days (**E**,**F**) of embryonic development. The proteins were characterized in three categories: biological process, cel-lular component, and molecular function. The 2, 6, and 13 days of embryonic development was compared with the 0, 2, and 6 days of embryonic development as control, respectively.

The results revealed differentially expressed proteins at 0, 2, 6, and 13 days of embryonic development in low and high cholesterol eggs such that 0 days was set as a control for two days of incubation, two days was set as a control for six days of incubation, and six days was used as a control for 13 days of incubation. In low cholesterol eggs, the two days of embryonic development had five differentially expressed proteins in biological processes, i.e., VTG-1, VTG-2, VTG-3, P01875, and OIH. The vitellogenin proteins were involved in lipid transport (GO:0006869) and lipid localization (GO:0010876); the OIH and P01875 showed involvement in response to mineralocorticoid (GO:0051385) and positive regulation of B cell proliferation (GO:0030890), respectively (Figure 16A, for detail, see Supplementary Table S1). The high cholesterol eggs have the same differentially expressed proteins at two days of embryonic development but regulated multiple biological processes. For example, the VTG-1, VTG-2, and VTG-3 were involved in lipid transport (GO:0006869) and lipid localization (GO: 0010876), and P01875 showed involvement in complement activation, classical pathway (GO:0006958), humoral immune response mediated by circulating immunoglobulin (GO:0002455), positive regulation of B cell proliferation (GO:0030890), and complement activation (GO:0006956). The OIH protein regulated response to mineralocorticoid (GO:0051385), response to corticosterone (GO:0051412), negative regulation of viral genome replication (GO:0045071), and negative regulation of viral life cycle (GO:1903901) (Figure 16B, for detail, see Supplementary Table S2). We detected seven differentially expressed proteins after six days of incubation in low cholesterol eggs namely, the VTG-1, VTG-2, VTG-3, IGLL1, P01875, TF, and Ig lambda chain V-1 region (P04210). The IGLL1 and P01875 were involved in different biological processes such as complement activation, classical pathway (GO:0006958), humoral immune response mediated by circulating immunoglobulin (GO:0002455), complement activation (GO:0006956), B cell-mediated immunity (GO:0019724), protein activation cascade (GO:0072376), and immunoglobulin mediated immune response (GO:0016064). The TF was involved in positive regulation of plasminogen activation (GO:0010756); IGLL1 and P04210 were associated with immunoglobulin production (GO:0002377), and production of molecular mediator of the immune response (GO:0002440); the vitellogenin proteins functioned in lipid transport and lipid localization processes (Figure 16C, for detail, see Supplementary Table S3).

At six days of incubation, the high cholesterol eggs showed eight differentially expressed proteins. The vitellogenin proteins were related to the lipid transport and lipid localization; P04210 and P01875 characterized complement activation, classical pathway (GO:0006958), humoral immune response mediated by circulating immunoglobulin (GO:0002455), complement activation (GO:0006956), and protein activation cascade (GO:0072376); the BG regulated production of molecular mediator of the immune responses (GO:0002440) with the help of IGLL1 and P04210. The BG was also involved in the regulation of symbiosis (GO:0043903) and response to bacterial lipopeptide (GO:0070339) in high cholesterol eggs (Figure 16D, for detail, see Supplementary Table S4). The 13 days of embryonic development showed seven differentially expressed protein in both egg types that defined the biological processes of the immune response (GO:0006955), activation of the immune response (GO:0002253), production of molecular mediator of the immune response (GO:0002440), lipid localization (GO:0010876), and lipid transport (GO:0006869). The genes expression profiles at 13 days were higher in high cholesterol compared to low cholesterol eggs in the above biological processes as shown in Figure 16E,F. (For detail, see Supplementary Tables S5 and S6).

### 3.4. GO Annotation of Differentially Expressed Proteins in Cellular Component

The GO annotation of differentially expressed proteins in the cellular component after 0–13 days of incubation revealed six differentially expressed proteins in low cholesterol eggs (Supplementary Tables S1, S3 and S5) and 5 proteins in high cholesterol eggs respectively (Supplementary Tables S2, S4 and S6). The ovalbumin was not spotted in a significant amount in high cholesterol eggs at 2 and 6 days of incubation (Supplementary Tables S2 and S4) while detected in low cholesterol eggs (Supplementary Tables S1 and S3). Similarly, the BG protein was not expressed significantly at two days of embryonic development in low cholesterol eggs (Supplementary Tables S1 and S3) while expressed in high cholesterol eggs (Supplementary Table S4). The common cellular components that were regulated during the embryonic development (0–13 days) by TF, IGLL1, P04210, P01875, and OIH proteins in both egg types were extracellular space (GO:0005615), extracellular region (GO:0005576), and extracellular region part (GO: 0044421), anchored component of the plasma membrane (GO:0046658), and anchored component of membrane (GO:0031225) (Figure 16A–F, For detail see Supplementary Tables S1–S6).

Notably, the VMO protein was involved in an extracellular exosome (GO:0070062), extracellular vesicle (GO:1903561), extracellular organelle (GO:0043230), and membrane-bounded vesicle (GO:0031988) at two days of incubation in both egg types but was not detected at 6 and 13 days of incubation in both eggs that indicated redundant nature of VMO at later stages of development (Supplementary Tables S1–S6). Overall, the two and six-day embryonic development showed variation in cellular components while the 13 days of incubation was similar in both egg types as shown in Figure 16E,F, and Supplementary Tables S5 and S6.

### 3.5. GO Annotation of Differentially Expressed Proteins in Molecular Function

At 2 and 6 days of embryonic development, the three vitellogenin proteins were associated with nutrient reservoir activity (GO:0045735), lipid transporter activity (GO: 0005319), substrate-specific transporter activity (GO:0022892), and transporter activity (GO:0005215) in both egg types (Figure 16A–D; Supplementary Tables S1–S4). The high and low cholesterol eggs differentially expressed proteins showed similar molecular functions at two days of incubation but exhibited variation at six days of incubation. Importantly, the BG protein was associated with various molecular functions such as lipoteichoic acid receptor activity (GO:0070892), low-density lipoprotein receptor activity (GO:0005041), high-density lipoprotein particle binding (GO:0008035), pattern recognition receptor activity (GO:0038187), signaling pattern recognition receptor activity (GO:0008329), and lipoprotein particle receptor activity (GO:0030228) (Figure 16C–F; Supplementary Tables S3–S6). In low cholesterol eggs, the OIH was involved in the molecular function of potassium channel inhibitor activity (GO:0019870), potassium channel regulator activity (GO:0015459), ion channel inhibitor activity (GO:0008200), channel inhibitor activity (GO:0016248), and protease binding (GO:0002020) at 2 and six days of incubation (Supplementary Tables S1 and S3) while at 13 days of embryonic development, the OIH expression was low and only regulated the potassium channel inhibitor activity (GO:0019870) (Supplementary Table S5). At 13 days of embryonic development, both egg types showed seven differentially expressed proteins that were involved in similar molecular functions such as nutrient reservoir activity, lipid transporter activity, antigen binding, lipoteichoic acid receptor activity, and low-density lipoprotein receptor activity (Figure 16E,F; Supplementary Tables S5 and S6). In summary, the 2 and 6 days of embryonic development showed variation in biological processes, cellular components, and molecular functions in both egg types, with high cholesterol eggs showing a higher level of gene expression relative to low cholesterol eggs.

## 4. Discussion

Protein expression profiling by 2-D gel electrophoresis is extensively used to determine the egg protein abundance and composition. Fertilized eggs had been recently explored that revealed cholesterol has positive impact on egg yolk protein abundance during embryogenesis [19]. To date, different factors such as temperature, storage period, and incubation time have been considered as effectors that modulated the egg yolk proteins abundance and expression level [30,36,37]. However, the effects of cholesterol concentration on the changes of egg yolk proteins remained unknown. In the present study, the egg quality characteristics between low and high-cholesterol egg yolks were, and the yolk protein intensities were determined at different incubation days. Through the study of low and high cholesterol eggs, the results revealed that vitellogenin proteins are the most abundant egg yolk protein in both egg types and showed a close resemblance with each other. VTG-1 and VTG-2 proteins expression significantly varied at 2 and 13 days of incubation in low and high cholesterol eggs respectively while VTG-3 protein intensities were significant at 2 and 6 days of incubation in low cholesterol relative to high cholesterol eggs. To date, the effect of cholesterol concentration on vitellogenin was not studied and we revealed that yolk cholesterol altered vitellogenin proteins intensity significantly. A previous study revealed that vitellogenin proteins facilitated embryo development and were involved in oocytes differentiation [38]. In present study, the VTG-1, VTG-2, and VTG-3 proteins showed a decrease in experimental pI/MW values. The reason behind lower experimental pI/MW values indicated vitellogenin proteins degradation by matrix metalloproteinase enzyme or endogenous proteases [30] and these degraded products might be used as a source of nutrient for the embryo during development. Our results are in accordance with the several reports where the authors summarized variation in MW and pI values of egg white and yolk proteins during incubation [29,37,39]. In our previous study, the results showed a significant decrease in vitellogenin proteins expression level at 18-days of incubation [19] while in the present study the results revealed a lower expression of VTG-3 and high expression of VTG-2 at 13 days of incubation. These findings indicated that protein intensities changed depending on the specific stage of embryo development such as 18-days of embryogenesis indicated the embryo hatching stage [2] while 13 days of embryonic development specified the embryo completion stage [25]. Importantly, our results found the TF protein in egg yolk which is one of the most abundant egg white protein [1] that showed its role in embryonic cartilage formation and embryo protection against pathogens during embryogenesis [38]. The TF spots were significantly expressed in high cholesterol than low cholesterol eggs that indicated the TF intensity is highly altered by cholesterol concentration. Similarly, our results detected significant expression of OIH in high cholesterol than low cholesterol eggs. The OIH protein was widely detected in the egg white of chicken, ostrich, and quail [40] and are associated with egg shelf-life [1,41]. The OIH was detected at a low level in fertilized egg white during embryogenesis [29,36] while we found a high abundance of OIH in egg yolk. Therefore, we concluded that the cholesterol concentration increased the OIH intensity that help in the protection of the embryo from pathogen because OIH belongs to the protease inhibitors family and has antimicrobial activity [41]. The SA is known as a food allergen and is involved in embryo development [42]. Wang and Wu, (2014) detected two SA spots in egg white with a decrease in abundance during embryogenesis [36]. In the present study, five spots of SA in egg yolk were detected which expression was significantly altered by cholesterol concentration in both egg types at two and six days of incubation. The immunoglobulin-Y has 2 heavy and 2 light chains and is synthesized by mature B cells in birds [43]. The immunoglobulin accumulated in the egg yolk by blood transportation and protects the developing embryo [44]. We identified Ig gamma in seven spots, IGLL in three spots, and P01875 in two spots in egg yolks with a high level of expression in high cholesterol relative to low cholesterol eggs, which indicated passive immunization for embryo during embryogenesis [45]. The immunoglobulin molecules also showed an increase in experimental MW compared to theoretical values, which likely indicates the phosphorylation of the native protein [46]. We detected a single spot of PIT-54 protein that can be used to differentiate chicken from duck and quail egg products [38]. The PIT-54 protein had antibacterial and antioxidant properties [47] and is involved in the protection of the embryo against pathogens during embryogenesis [19]. In our study, the VMO showed high expression in low cholesterol compared to the high cholesterol eggs and showed lower experimental MW than theoretical value. The VMO protein separates the yolk from the egg white and is involved in the formation of protective fibrous layers in avian eggs [48] and could elicit hemagglutination [49]. However, its functional role in chicken embryonic development is not widely studied and remained uncertain. In addition, our study revealed that most of the yolk proteins during embryogenesis were involved in food reservation, protection of the embryo, and transportation activities, such as the VTG-1, VTG-2, and VTG-3 were involved in lipid transport and nutrient reservation activity.

Vitellogenin has been considered as the main nutrient source for developing embryo and is synthesized by the liver in response to estrogenic activation [50]. The antibodies Ig gamma, IGLC, P01875, and IGLL provide an efficient humoral immune response against the pathogens similar to the previous report where these proteins play a vital role in antibodies mediated immune responses [51]. The TF regulated the multiple extracellular matrix disassembly pathways and are associated with endochondral bone formation in developing chick embryos while the OIH has physiological importance for embryo development and a decrease the decomposition of yolk nutrients because OIH is involved in response to mineralocorticoid, corticosteroid, glucocorticoid, and ketone physiological reaction before hatching. In summary, the biological processes, cellular components, molecular functions, and the observed differences between low- and high-cholesterol egg yolks during embryogenesis will possibly pave the way for future research on yolk cholesterol concerning incubation time, temperature, and environmental conditions.

## 5. Conclusions

Cholesterol is essential for embryo development and an important entity for proteins and lipid synthesis during embryogenesis. The effects of cholesterol concentration on yolk proteins are not well characterized and our present work quantifies the alteration in abundance and intensities of low and high-fertilized egg yolk plasma proteins during embryonic development. The study revealed that the intensities of the protein varied because of low and high cholesterol concentration and the majority of yolk protein is associated with the cholesterol efflux pathway. Most of the egg yolk proteins significantly expressed in high cholesterol relative to low cholesterol egg. The egg yolk proteins such as VTG1, VTG2, and VTG3 were involved in lipid localization and lipid transportation that facilitates nutrient transport and chondrogenesis of developing embryos. The low and high cholesterol eggs showed similar yolk proteins profiles but different expression levels. Thus, the concentration of yolk cholesterol creates a biological significance and protective nutritional environment for the embryo from the initial stages of incubation until hatching. It will be of great interest to reveal that the egg yolk protein can enhance cholesterol synthesis or an impaired cholesterol metabolism during egg formation and embryo development.

## Figures and Tables

**Table 1:** The table enlisted all of the proteins detected in low and high cholesterol groups with their theoretical and experimental pI and MW values, accession numbers, score, and sequence coverage.

Spots	Identification Results	NCBI Accession	Score	Sequence Coverage (%)	Theoretical pI/MW (kDa)	Experimental pI/MW (kDa)
1	Ovotransferrin BB type	71274077	962	22	7.08/79.6	7.1/75
2	Ovotransferrin BB type	71274077	1148	23	7.08/79.6	7.2/75
3	Ovotransferrin BB type	71274077	1222	23	7.08/79.6	7.4/75
4	Ovotransferrin BB type	71274077	484	14	7.08/79.6	7.6/75
5	Ovotransferrin BB type	71274077	857	20	7.08/79.6	7.8/75
62	Ovotransferrin BB type	71274077	764	27	7.08/79.6	7.1/77
6	Ig gamma chain (clone-36) chicken	63524	384	23	6.84/54.5	7.5/68
14	Ig gamma chain (clone-36) chicken	63524	357	22	6.84/54.5	6.1/70
21	Ig gamma chain (clone-36) chicken	63524	156	22	6.84/54.5	6.0/57
61	Ig gamma chain (clone-36) chicken	63524	473	24	6.84/54.5	7.8/62
64	Ig gamma chain (clone-36) chicken	63524	268	17	6.84/54.5	6.0/63
66	Ig gamma chain (clone-36) chicken	63524	255	24	6.84/54.5	7.0/70
67	Ig gamma chain (clone-36) chicken	63524	246	18	6.84/54.5	6.5/68
72	Ig gamma chain (clone-36) chicken	63524	198	24	6.84/54.5	7.2/35
73	Ig gamma chain (clone-36) chicken	63524	175	26	6.84/54.5	7.4/35
7	Immunoglobulin-Y heavy chain constant region	614458442	376	18	6.11/43.5	7.0/72
8	Immunoglobulin-Y heavy chain constant region	614458442	479	18	6.11/43.5	7.0/72
51	Immunoglobulin lambda light chain precursor	266634462	65	11	5.66/23.2	5.6/23
53	Immunoglobulin lambda light chain precursor	266634462	65	11	5.66/23.2	5.6/23
54	Immunoglobulin lambda light chain precursor	266634462	130	11	5.66/23.2	5.6/23
11	Ovoinhibitor	212485	275	25	6.16/54.4	5.6/68
12	Ovoinhibitor	212485	100	21	6.16/54.4	5.8/68
13	Ovoinhibitor	212485	80	17	6.16/54.4	5.8/68
15	Ovoinhibitor	212485	102	24	6.16/54.4	5.8/64
16	Ovoinhibitor	212485	62	18	6.16/54.4	6.0/64
17	Ovoinhibitor	212485	61	22	6.16/54.4	6.0/64
20	Ovoinhibitor	212485	44	18	6.16/54.4	5.8/62
52	Ovoinhibitor	212485	73	17	6.16/54.4	5.5/23
63	Ovoinhibitor	212485	65	21	6.16/54.4	5.8/62
23	Vitellogenin-1 precursor	1871444	226	5	9.16/212.6	5.2/40
24	Vitellogenin-1 precursor	1871444	601	4	9.16/212.6	5.2/40
25	Vitellogenin-1 precursor	1871444	618	4	9.16/212.6	5.4/40
26	Vitellogenin-1 precursor	1871444	273	3	9.16/212.6	5.4/40
27	Vitellogenin-1 precursor	1871444	180	2	9.16/212.6	5.6/40
50	Vitellogenin-1 precursor	1871444	83	3	9.16/212.6	5.8/40
28	Vitellogenin-2	63887	183	1	9.23/206.7	5.5/36
29	Vitellogenin-2	63887	667	5	9.23/206.7	5.5/36
30	Vitellogenin-2	63887	159	2	9.23/206.7	5.8/36
31	Vitellogenin-2	63887	293	2	9.23/206.7	6.0/36
32	Vitellogenin-2	63887	579	4	9.23/206.7	5.5/33
33	Vitellogenin-2	63887	356	3	9.23/206.7	5.6/33
34	Vitellogenin-2	63887	243	2	9.23/206.7	5.8/33
35	Vitellogenin-2	63887	361	3	9.23/206.7	6.0/33
36	Vitellogenin-2	63887	264	1	9.23/206.7	6.2/33
41	Vitellogenin-2	63887	224	5	9.23/206.7	6.2/28
43	Vitellogenin-2	63887	266	5	9.23/206.7	6.2/28
44	Vitellogenin-2	63887	76	5	9.23/206.7	6.2/28
45	Vitellogenin-2	63887	261	5	9.23/206.7	6.2/28
46	Vitellogenin-2	63887	365	4	9.23/206.7	6.2/26
65	Vitellogenin-2	63887	391	5	9.23/206.7	7.2/28
48	Vitellogenin-3	971408444	157	4	8.93/193.3	5.4/28
49	Vitellogenin-3	971408444	112	2	8.93/193.3	5.6/28
74	Vitellogenin-3	971408444	181	3	8.93/193.3	6.8/45
56	Vitelline membrane outer layer protein-1	268370086	358	33	5.21/21.5	3.8/20
57	Vitelline membrane outer layer protein-1	268370086	496	33	5.21/21.5	3.8/20
58	Vitelline membrane outer layer protein-1	268370086	389	33	5.21/21.5	4.0/20
59	Vitelline membrane outer layer protein-1	268370086	284	33	5.21/21.5	4.0/20
10	Serum albumin	63748	327	16	5.51/71.9	5.0/70
22	Serum albumin	63748	279	16	5.51/71.9	4.6/50
37	Serum albumin	63748	45	9	5.51/71.9	4.6/33
38	Serum albumin	63748	42	13	5.51/71.9	5.0/34
39	Serum albumin	63748	250	6	5.51/71.9	5.0/34
9	PIT-54	13434994	327	36	4.61/52.7	3.9/68
70	Beta-2-glycoprotein-1 precursor	487439524	304	31	8.6/40.1	7.0/48.5
71	Beta-2-glycoprotein-1 precursor	487439524	373	31	8.6/40.1	7.1/48.5

**Table 2:** The table showed the egg quality parameters and cholesterol amount determined in egg yolk in low and high cholesterol eggs.

Parameters	Low Cholesterol Egg	High Cholesterol Egg
Cholesterol concentration (mg/g/egg)	30.07 ± 1.147	40.27 ± 1.022
Egg weight (g)	45.30 ± 0.421	46.00 ± 0.487
Yolk weight (g)	13.57 ± 0.115	14.02 ± 0.326
Egg shape index	1.290 ± 0.005	1.297 ± 0.003
Egg strength (mm)	3.760 ± 0.078	3.957 ± 0.0866
Haugh unit	66.01 ± 0.904	65.49 ± 2.133
Shell thickness (mm)	38.26 ± 0.178	38.46 ± 0.283
Specific gravity (gcm^−3^)	13.89 ± 0.170	13.98 ± 0.018
Albumin height (mm)	4.150 ± 0.095	4.487 ± 0.132
Color	3.677 ± 0.117	3.783 ± 0.187

## Supplementary Materials

The following are available online at https://www.mdpi.com/2076-2615/11/3/744/s1. Supplementary Figure S1. The SA, IGLL, P01875, PIT54, and VMO protein intensities after 0, 2, 6, and 13 days of incubation are shown in high cholesterol eggs. The column profiles of SA (10, 22, 37–39), IGLL (51, 53, 54), P01875 (7, 8), PIT54 (9) and VMO (56) are shown as bar charts with significant differences. The values are from three independent replicates and the error bars represent the standard deviation. Supplementary Figure S2. The changes in the intensity of SA, IGLL, P01875, PIT54, and VMO proteins at 0, 2, 6, and 13 days of incubation are shown in low cholesterol eggs. The column profiles of SA (10, 22, 37–39), IGLL (51, 53, 54), P01875 (7, 8), PIT54 (9) and VMO (56) are indicated as bar charts with significant differences. The values are from three independent replicates and the error bars represent the standard deviation. Supplementary Table S1. Go annotation of differentially expressed proteins in biological process, cellular component, and molecular function after two days of incubation compared to the control (0 days) in low cholesterol eggs. Supplementary Table S2. Go annotation of differentially expressed proteins in biological process, cellular component, and molecular function after two days of incubation compared to the control (0 days) in high cholesterol eggs. Supplementary Table S3. Go annotation of differentially expressed proteins in biological process, cellular component, and molecular function after six days of incubation compared to the control (two days) in low cholesterol eggs. Supplementary Table S4. Go annotation of differentially expressed proteins in biological process, cellular component, and molecular function after six days of incubation compared to the control (two days) in high cholesterol eggs. Supplementary Table S5. Go annotation of differentially expressed proteins in biological process, cellular component, and molecular function after 13 days of incubation compared to the control (six days) in low cholesterol eggs. Supplementary Table S6. Go annotation of differentially expressed proteins in biological process, cellular component, and molecular function after 13 days of incubation compared to the control (six days) in high cholesterol eggs.

## References

[1] Guérin-Dubiard C., Pasco M., Mollé D., Désert C., Croguennec T., Nau F. (2006). Proteomic analysis of hen egg white. J. Agric. Food Chem..

[2] Moran E.T. (2007). Nutrition of the developing embryo and hatchling. Poult. Sci..

[3] Muramatsu T., Hiramoto K., Koshi N., Okumura J., Miyoshi S., Mitsumoto T. (1990). Importance of albumen content in whole-body protein synthesis of the chicken embryo during incubation. Br. Poult. Sci..

[4] Burley R. (1989). The Avian Egg: Chemistry and Biology.

[5] Mann K., Mann M. (2008). The chicken egg yolk plasma and granule proteomes. Proteomics.

[6] Li C., Geng F., Huang X., Ma M., Zhang X. (2014). Phosvitin phosphorus is involved in chicken embryo bone formation through dephosphorylation. Poult. Sci..

[7] Wallace R.A., Hoch K.L., Carnevali O. (1990). Placement of small lipovitellin subunits within the vitellogenin precursor in Xenopus laevis. J. Mol. Biol..

[8] McClance R. (1998). Fatty Acids: Seventh Supplement to the Fifth Edition of McCance and Widdowson’s The Composition of Foods.

[9] Andersen C.J., Blesso C.N., Lee J., Barona J., Shah D., Thomas M.J., Fernandez M.L. (2013). Egg consumption modulates HDL lipid composition and increases the cholesterol-accepting capacity of serum in metabolic syndrome. Lipids.

[10] Omole J., Ighodaro O. (2012). Comparative studies of the effects of egg yolk, oats, apple, and wheat bran on serum lipid profile of wistar rats. ISRN Nutr..

[11] Panda A., Reddy M., Rao S.R., Praharaj N. (2003). Production performance, serum/yolk cholesterol and immune competence of white leghorn layers as influenced by dietary supplementation with probiotic. Trop. Anim. Health Prod..

[12] Yang P., Tian Y., Sun G., Jiang R., Han R., Kang X. (2013). Deposition rule of yolk cholesterol in two different breeds of laying hens. Genet. Mol. Res..

[13] Dikmen B.Y., Sahan U. (2007). Correlations between breeder age, egg cholesterol content, blood cholesterol level and hatchability of broiler breeders. Br. Poult. Sci..

[14] Zhou L., Shi Y., Guo R., Liang M., Zhu X., Wang C. (2014). Digital gene-expression profiling analysis of the cholesterol-lowering effects of alfalfa saponin extract on laying hens. PLoS ONE.

[15] Dietschy J.M., Turley S.D., Spady D.K. (1993). Role of liver in the maintenance of cholesterol and low density lipoprotein homeostasis in different animal species, including humans. J. Lipid Res..

[16] Battaile K.P., Steiner R.D. (2000). Smith-Lemli-Opitz syndrome: The first malformation syndrome associated with defective cholesterol synthesis. Mol. Genet. Metab..

[17] Porter F.D. (2000). RSH/Smith–Lemli–Opitz syndrome: A multiple congenital anomaly/mental retardation syndrome due to an inborn error of cholesterol biosynthesis. Mol. Genet. Metab..

[18] Bottjer K.P., Weinstein P.P., Thompson M.J. (1985). Effects of an azasteroid on growth, development and reproduction of the free-living nematodes Caenorhabditis briggsae and Panagrellus redivivus. Comp. Biochem. Physiol. B Comp. Biochem..

[19] Zhu W., Zhang J., He K., Geng Z., Chen X. (2020). Proteomic analysis of fertilized egg yolk proteins during embryonic development. Poult. Sci..

[20] Fauziah C., Zaibunnisa A., Osman H., Wan Aida W. (2016). Physicochemical analysis of cholesterol-reduced egg yolk powder and its application in mayonnaise. Int. Food Res. J..

[21] Nezil F.A., Bloom M. (1992). Combined influence of cholesterol and synthetic amphiphillic peptides upon bilayer thickness in model membranes. Biophys. J..

[22] Rothberg K.G., Heuser J.E., Donzell W.C., Ying Y.-S., Glenney J.R., Anderson R.G. (1992). Caveolin, a protein component of caveolae membrane coats. Cell.

[23] Foster L.J., De Hoog C.L., Mann M. (2003). Unbiased quantitative proteomics of lipid rafts reveals high specificity for signaling factors. Proc. Natl. Acad. Sci. USA.

[24] Cole T.J., Blendy J.A., Monaghan A.P., Krieglstein K., Schmid W., Aguzzi A., Fantuzzi G., Hummler E., Unsicker K., Schütz G. (1995). Targeted disruption of the glucocorticoid receptor gene blocks adrenergic chromaffin cell development and severely retards lung maturation. Genes Dev..

[25] Cordeiro C.M., Hincke M.T. (2016). Quantitative proteomics analysis of eggshell membrane proteins during chick embryonic development. J. Proteom..

[26] Berger S., Bleich M., Schmid W., Cole T.J., Peters J., Watanabe H., Kriz W., Warth R., Greger R., Schütz G. (1998). Mineralocorticoid receptor knockout mice: Pathophysiology of Na+ metabolism. Proc. Natl. Acad. Sci. USA.

[27] Pratt H.P. (1982). Preimplantation mouse embryos synthesize membrane sterols. Dev. Biol..

[28] Larsen W. (2001). Human Embryology.

[29] Qiu N., Ma M., Cai Z., Jin Y., Huang X., Huang Q., Sun S. (2012). Proteomic analysis of egg white proteins during the early phase of embryonic development. J. Proteom..

[30] Rehault-Godbert S., Mann K., Bourin M., Brionne A., Nys Y. (2014). Effect of embryonic development on the chicken egg yolk plasma proteome after 12 days of incubation. J. Agric. Food Chem..

[31] Liu Y., Qiu N., Ma M. (2015). Comparative proteomic analysis of egg white proteins during the rapid embryonic growth period by combinatorial peptide ligand libraries. Poult. Sci..

[32] Chen X., Zhu W., Du Y., Liu X., Geng Z. (2019). Genetic Parameters for Yolk Cholesterol and Transcriptional Evidence Indicate a Role of Lipoprotein Lipase in the Cholesterol Metabolism of the Chinese Wenchang Chicken. Front. Genet..

[33] Naveena B., Faustman C., Tatiyaborworntham N., Yin S., Ramanathan R., Mancini R. (2010). Detection of 4-hydroxy-2-nonenal adducts of turkey and chicken myoglobins using mass spectrometry. Food Chem..

[34] Qian X., Yang Y., Lee S.W., Caballes M.J., Alamu O.S. (2020). Cooling Performance Analysis of the Lab-Scale Hybrid Oyster Refrigeration System. Processes.

[35] Qian X., Lee S.W. The design and analysis of energy efficient building envelopes for the commercial buildings by mixed-level factorial design and statistical methods. Proceedings of the ASEE Middle Atlantic American Society of Engineering Education.

[36] Wang J., Wu J. (2014). Proteomic analysis of fertilized egg white during early incubation. EuPA Open Proteom..

[37] Qiu N., Ma M., Zhao L., Liu W., Li Y., Mine Y. (2012). Comparative proteomic analysis of egg white proteins under various storage temperatures. J. Agric. Food Chem..

[38] Liu Y., Qiu N., Gao D., Ma M. (2018). Comparative proteomic analysis of chicken, duck, and quail egg yolks. Int. J. Food Prop..

[39] Meng Y., Sun H., Qiu N., Geng F., Zhu F., Li S., Huo Y. (2019). Comparative proteomic analysis of hen egg yolk plasma proteins during embryonic development. J. Food Biochem..

[40] Hu S., Qiu N., Liu Y., Zhao H., Gao D., Song R., Ma M. (2016). Identification and comparative proteomic study of quail and duck egg white protein using 2-dimensional gel electrophoresis and matrix-assisted laser desorption/ionization time-of-flight tandem mass spectrometry analysis. Poult. Sci..

[41] Bourin M., Gautron J., Berges M., Attucci S., Le Blay G., Labas V., Nys Y., Rehault-Godbert S. (2011). Antimicrobial potential of egg yolk ovoinhibitor, a multidomain Kazal-like inhibitor of chicken egg. J. Agric. Food Chem..

[42] Quirce S., Maranon F., Umpierrez A., De Las Heras M., Fernández-Caldas E., Sastre J. (2001). Chicken serum albumin (Gal d 5*) is a partially heat-labile inhalant and food allergen implicated in the bird-egg syndrome. Allergy.

[43] Chalghoumi R., Beckers Y., Portetelle D., Théwis A. (2009). Hen egg yolk antibodies (IgY), production and use for passive immunization against bacterial enteric infections in chicken: A review. Biotechnol. Agron. Soc. Environ..

[44] Hamal K., Burgess S.C., Pevzner I., Erf G. (2006). Maternal antibody transfer from dams to their egg yolks, egg whites, and chicks in meat lines of chickens. Poult. Sci..

[45] Hasselquist D., Nilsson J.-Å. (2009). Maternal transfer of antibodies in vertebrates: Trans-generational effects on offspring immunity. Philos. Trans. R. Soc. B Biol. Sci..

[46] Gao D., Qiu N., Liu Y., Ma M. (2017). Comparative proteome analysis of egg yolk plasma proteins during storage. J. Sci. Food Agric..

[47] Georgieva T.M., Koinarski V., Urumova V., Marutsov P., Christov T., Nikolov J., Chaprazov T., Walshe K., Karov R., Georgiev I. (2010). Effects of Escherichia coli infection and Eimeria tenella invasion on blood concentrations of some positive acute phase proteins (haptoglobin (PIT 54), fibrinogen and ceruloplasmin) in chickens. Revue Med. Vet..

[48] Lim W., Song G. (2015). Differential expression of vitelline membrane outer layer protein 1: Hormonal regulation of expression in the oviduct and in ovarian carcinomas from laying hens. Mol. Cell. Endocrinol..

[49] Kido S., Doi Y., Kim F., Morishita E., Narita H., Kanaya S., Ohkubo T., Nishikawa K., Yao T., Ooi T. (1995). Characterization of vitelline membrane outer layer protein I, VMO-I: Amino acid sequence and structural stability. J. Biochem..

[50] Wang S., Smith D.E., Williams D.L. (1983). Purification of avian vitellogenin III: Comparison with vitellogenins I and II. Biochemistry.

[51] Tian Z., Zhang X. (2012). Progress on research of chicken IgY antibody-FcRY receptor combination and transfer. J. Recept. Signal Transduct..

