# Supplementary material for: Comparative Yolk Proteomic Analysis of Fertilized Low and High Cholesterol Eggs during Embryonic Development
Source: Animals (Basel). 2021 Mar 9;11(3):744. doi: 10.3390/ani11030744 (PMC8035655; doi:10.3390/ani11030744)
Supplement: Supplementary file 1 [file animals-11-00744-s001.zip › Supplementary Tables S1-S6/Supplementary Table S1.docx]

| **GO annotation of differentially expressed proteins in biological process after 2-days of incubation compared to the control (0 days) in low cholesterol egg** | | | |
| --- | --- | --- | --- |
| **Serial number** | **Annotation** | **Differentially expressed protein^1^** | **P-value** |
| GO:0006869 | lipid transport | VTG1, VTG2, VTG3 | 0.0001 |
| GO:0010876 | lipid localization | VTG1, VTG2, VTG3 | 0.0001 |
| GO:0010756 | positive regulation of plasminogen activation | TF | 0.0006 |
| GO:0090091 | positive regulation of extracellular matrix disassembly | TF | 0.0009 |
| GO:0010755 | regulation of plasminogen activation | TF | 0.002 |
| GO:0010715 | regulation of extracellular matrix disassembly | TF | 0.002 |
| GO:1900025 | negative regulation of substrate adhesion-dependent cell spreading | TF | 0.002 |
| GO:0051385 | response to mineralocorticoid | OIH | 0.002 |
| GO:0051412 | response to corticosterone | OIH | 0.002 |
| GO:0097286 | iron ion import | TF | 0.002 |
| GO:0030890 | positive regulation of B cell proliferation | P01875 | 0.003 |
|  | | | |
| **Go annotation of differentially expressed proteins in cellular component after 2-days of incubation compared to the control (0 days)** | | | |
| Serial number | Annotation | Differentially expressed protein^1^ | P-value |
| GO:0005615 | extracellular space | TF, ALB, OIH, IGLL1, VMO1 | 0.0000008 |
| GO:0005576 | extracellular region | TF, P01875, ALB, OIH, IGLL1, VMO1 | 0.000008 |
| GO:0044421 | extracellular region part | TF, ALB, OIH, IGLL1, VMO1 | 0.0001 |
| GO:0046658 | anchored component of plasma membrane | TF | 0.006 |
| GO:0031225 | anchored component of membrane | TF | 0.01 |
| GO:0065010 | extracellular membrane-bounded organelle | TF, VMO1 | 0.06 |
| GO:0070062 | extracellular exosome | TF, VMO1 | 0.07 |
| GO:1903561 | extracellular vesicle | TF, VMO1 | 0.07 |
| GO:0043230 | extracellular organelle | TF, VMO1 | 0.07 |
| GO:0031988 | membrane-bounded vesicle | TF, VMO1 | 0.09 |
|  | | | |
| **Go annotation of differentially expressed proteins in molecular function after 2-days of incubation compared to the control (0 days)** | | | |
| **Serial number** | **Annotation** | **Differentially expressed protein^1^** | **P-value** |
| GO:0045735 | nutrient reservoir activity | VTG2, VTG3 | 0.0000002 |
| GO:0005319 | lipid transporter activity | VTG1, VTG2, VTG3 | 0.000002 |
| GO:0019870 | potassium channel inhibitor activity | OIH | 0.001 |
| GO:0022892 | substrate-specific transporter activity | VTG1, VTG2, VTG3 | 0.003 |
| GO:0015459 | potassium channel regulator activity | OIH | 0.006 |
| GO:0008200 | ion channel inhibitor activity | OIH | 0.007 |
| GO:0016248 | channel inhibitor activity | OIH | 0.007 |
| GO:0005215 | transporter activity | VTG1, VTG2, VTG3 | 0.008 |
| GO:0003823 | antigen binding | P01875 | 0.01 |
| GO:0002020 | protease binding | OIH | 0.01 |
|  | | | |
| ^1^Differentially expressed protein. VTG1, VTG2, VTG3, OIH, IGLL1, P01875, ALB, TF, and VMO1 represent the gene names of vitellogenin-Ⅰ precursor, vitellogenin-Ⅱ, vitellogenin-Ⅲ, ovoinhibitor, immunoglobulin lambda light chain precursor, immunoglobulin Y heavy chain constant region, ovalbumin, ovotransferrin, and vitelline membrane outer layer protein respectively. List only the top 10-12 annotations for P values. | | | |
