# Supplementary material for: Comparative Yolk Proteomic Analysis of Fertilized Low and High Cholesterol Eggs during Embryonic Development
Source: Animals (Basel). 2021 Mar 9;11(3):744. doi: 10.3390/ani11030744 (PMC8035655; doi:10.3390/ani11030744)
Supplement: Supplementary file 1 [file animals-11-00744-s001.zip › Supplementary Tables S1-S6/Supplementary Table S2.docx]

| **Go annotation of differentially expressed proteins in biological process after 2-days of incubation compared to the control (0 days) in high cholesterol egg** | | | |
| --- | --- | --- | --- |
| **Serial number** | **Annotation** | **Differentially expressed protein^1^** | **P-value** |
| GO:0006869 | lipid transport | VTG1, VTG2, VTG3 | 0.00006 |
| GO:0010876 | lipid localization | VTG1, VTG2, VTG3 | 0.00008 |
| GO:0051385 | response to mineralocorticoid | OIH | 0.002 |
| GO:0051412 | response to corticosterone | OIH | 0.002 |
| GO:0006958 | complement activation classical pathway | P01875 | 0.002 |
| GO:0002455 | humoral immune response mediated by circulating immunoglobulin | P01875 | 0.004 |
| GO:0045071 | negative regulation of viral genome replication | OIH | 0.006 |
| GO:0030890 | positive regulation of B cell proliferation | P01875 | 0.009 |
| GO:1903901 | negative regulation of viral life cycle | OIH | 0.005 |
| GO:0006956 | complement activation | P01875 | 0.005 |
| GO:0048525 | negative regulation of viral process | OIH | 0.005 |
|  | | | |
| **Go annotation of differentially expressed proteins in cellular component after 2-days of incubation compared to the control (0 days)** | | | |
| **Serial number** | **Annotation** | **Differentially expressed protein^1^** | **P-value** |
| GO:0005615 | extracellular space | ALB, OIH, IGLL1, VMO1 | 0.00001 |
| GO:0005576 | extracellular region | ALB, OIH, IGLL1, VMO1, P01875 | 0.00005 |
| GO:0044421 | extracellular region part | ALB, OIH, IGLL1, VMO1 | 0.0009 |
| GO:0065010 | extracellular membrane-bounded organelle | VMO1 | 0.31 |
| GO:0070062 | extracellular exosome | VMO1 | 0.32 |
| GO:1903561 | extracellular vesicle | VMO1 | 0.33 |
| GO:0043230 | extracellular organelle | VMO1 | 0.33 |
| GO:0031988 | membrane-bounded vesicle | VMO1 | 0.36 |
| GO:0031982 | vesicle | VMO1 | 0.45 |
| GO:0043234 | protein complex | OIH | 0.63 |
|  | | | |
| **Go annotation of differentially expressed proteins in molecular function after 2-days of incubation compared to the control (0 days)** | | | |
| **Serial number** | **Annotation** | **Differentially expressed protein^1^** | **P-value** |
| GO:0045735 | nutrient reservoir activity | VTG2, VTG3 | 0.0000001 |
| GO:0005319 | lipid transporter activity | VTG1, VTG2, VTG3 | 0.000001 |
| GO:0019870 | potassium channel inhibitor activity | OIH | 0.0009 |
| GO:0022892 | substrate-specific transporter activity | VTG2, VTG3, VTG1 | 0.001 |
| GO:0005215 | transporter activity | VTG3,VTG1, VTG2 | 0.004 |
| GO:0015459 | potassium channel regulator activity | OIH | 0.005 |
| GO:0008200 | ion channel inhibitor activity | OIH | 0.005 |
| GO:0016248 | channel inhibitor activity | OIH | 0.006 |
| GO:0003823 | antigen binding | P01875 | 0.009 |
| GO:0002020 | protease binding | OIH | 0.015 |
|  | | | |
| ^1^Differentially expressed protein. VTG1, VTG2, VTG3, OIH, IGLL1, P01875, and ALB represent the gene names of vitellogenin-1 precursor, vitellogenin-2, vitellogenin-3, ovoinhibitor, immunoglobulin lambda light chain precursor, immunoglobulin Y heavy chain constant region, and ovalbumin respectively. List only the top 10-12 annotations for P values. | | | |
