# Supplementary material for: Comparative Yolk Proteomic Analysis of Fertilized Low and High Cholesterol Eggs during Embryonic Development
Source: Animals (Basel). 2021 Mar 9;11(3):744. doi: 10.3390/ani11030744 (PMC8035655; doi:10.3390/ani11030744)
Supplement: Supplementary file 1 [file animals-11-00744-s001.zip › Supplementary Tables S1-S6/Supplementary Table S3.docx]

| **Go annotation of differentially expressed proteins in biological process after 6-days of incubation compared to the control (2-days) in low cholesterol egg** | | | |
| --- | --- | --- | --- |
| **Serial number** | **Annotation** | **Differentially expressed protein^1^** | **P-value** |
| GO:0006958 | complement activation, classical pathway | P04210, P01875 | 0.000005 |
| GO:0002455 | humoral immune response mediated by circulating immunoglobulin | P04210, P01875 | 0.00001 |
| GO:0006956 | complement activation | P04210, P01875 | 0.0001 |
| GO:0072376 | protein activation cascade | P04210, P01875 | 0.0001 |
| GO:0006869 | lipid transport | VTG1, VTG2, VTG3 | 0.0001 |
| GO:0016064 | immunoglobulin mediated immune response | P04210, P01875 | 0.0002 |
| GO:0010876 | lipid localization | VTG1, VTG2, VTG3 | 0.0002 |
| GO:0019724 | B cell mediated immunity | P04210, P01875 | 0.0002 |
| GO:0002377 | immunoglobulin production | IGLL1, P04210 | 0.0002 |
| GO:0006959 | humoral immune response | P04210, P01875 | 0.0004 |
| GO:0010756 | positive regulation of plasminogen activation | TF | 0.0006 |
| GO:0002449 | lymphocyte mediated immunity | P01875, P04210 | 0.0007 |
| GO:0002440 | production of molecular mediator of immune response | IGLL1, P04210 | 0.0007 |
|  | | | |
| **Go annotation of differentially expressed proteins in cellular component after 6-days of incubation compared to the control (2-days)** | | | |
| **Serial number** | **Annotation** | **Differentially expressed protein^1^** | **P-value** |
| GO:0005615 | extracellular space | TF, ALB, OIH, IGLL1, P04210 | 0.0000008 |
| GO:0005576 | extracellular region | TF, ALB, OIH, IGLL1, P04210, P01875 | 0.000008 |
| GO:0044421 | extracellular region part | TF, ALB, OIH, IGLL1, P04210 | 0.0001 |
| GO:0046658 | anchored component of plasma membrane | TF | 0.006 |
| GO:0031225 | anchored component of membrane | TF | 0.005 |
| GO:0009986 | cell surface | TF | 0.005 |
| GO:0031226 | intrinsic component of plasma membrane | TF | 0.004 |
| GO:0065010 | extracellular membrane-bounded organelle | TF | 0.004 |
| GO:0070062 | extracellular exosome | TF | < 0.05 |
| GO:1903561 | extracellular vesicle | TF | < 0.05 |
| GO:0043230 | extracellular organelle | TF | < 0.05 |
|  | | | |
| **Go annotation of differentially expressed proteins in molecular function after 6-days of incubation compared to the control (2-days)** | | | |
| **Serial number** | **Annotation** | **Differentially expressed protein^1^** | **P-value** |
| GO:0045735 | nutrient reservoir activity | VTG2, VTG3 | 0.0000003 |
| GO:0005319 | lipid transporter activity | VTG1, VTG2, VTG3 | 0.000004 |
| GO:0003823 | antigen binding | P04210, P01875 | 0.00008 |
| GO:0019870 | potassium channel inhibitor activity | OIH | 0.0001 |
| GO:0022892 | substrate-specific transporter activity | VTG1, VTG2, VTG3 | 0.0005 |
| GO:0015459 | potassium channel regulator activity | OIH | 0.007 |
| GO:0008200 | ion channel inhibitor activity | OIH | 0.008 |
| GO:0016248 | channel inhibitor activity | OIH | 0.008 |
| GO:0005215 | transporter activity | VTG1, VTG2, VTG3 | 0.006 |
| GO:0002020 | protease binding | OIH | 0.005 |
| GO:0004867 | serine-type endopeptidase inhibitor activity | OIH | 0.005 |
| GO:0016247 | channel regulator activity | OIH | 0.006 |
|  | | | |
| ^1^Differentially expressed protein. VTG1, VTG2, VTG3, OIH, IGLL1, P01875, ALB, TF, and VMO1 represent the gene names of vitellogenin-1 precursor, vitellogenin-2, vitellogenin-3, ovoinhibitor, immunoglobulin lambda light chain precursor, immunoglobulin Y heavy chain constant region, ovalbumin, ovotransferrin, and vitelline membrane outer layer protein respectively. List only the top 10-12 annotations for P values. | | | |
