# Supplementary material for: Comparative Yolk Proteomic Analysis of Fertilized Low and High Cholesterol Eggs during Embryonic Development
Source: Animals (Basel). 2021 Mar 9;11(3):744. doi: 10.3390/ani11030744 (PMC8035655; doi:10.3390/ani11030744)
Supplement: Supplementary file 1 [file animals-11-00744-s001.zip › Supplementary Tables S1-S6/Supplementary Table S6.docx]

| **Go annotation of differentially expressed proteins in biological process after 13-days of incubation compared to the control (6-days) in high cholesterol egg** | | | |
| --- | --- | --- | --- |
| **Serial number** | **Annotation** | **Differentially expressed protein^1^** | **P-value** |
| GO:0006869 | lipid transport | VTG1, VTG2, VTG3, BG | 0.000003 |
| GO:0010876 | lipid localization | VTG1, VTG2, VTG3, BG | 0.000004 |
| GO:0006958 | complement activation classical pathway | P04210, P01875 | 0.000005 |
| GO:0002440 | production of molecular mediator of immune response | IGLL1, P04210, BG | 0.000007 |
| GO:0002455 | humoral immune response mediated by circulating immunoglobulin | P01875, P04210 | 0.00001 |
| GO:0006956 | complement activation | P01875, P04210 | 0.0001 |
| GO:0002253 | activation of immune response | P01875, P04210, BG | 0.0001 |
| GO:0072376 | protein activation cascade | P04210, P01875 | 0.0001 |
| GO:0006955 | immune response | IGLL1, P04210, P01875, BG | 0.0001 |
| GO:0016064 | immunoglobulin mediated immune response | P01875, P04210 | 0.0002 |
| GO:0019724 | B cell mediated immunity | P01875, P04210 | 0.0002 |
| GO:0002252 | immune effector process | P04210, P01875, BG | 0.0002 |
| GO:0045087 | innate immune response | P04210, P01875, BG | 0.0002 |
|  | | | |
| **Go annotation of differentially expressed proteins in cellular component after 13-days of incubation compared to the control (6-days)** | | | |
| **Serial number** | **Annotation** | **Differentially expressed protein^1^** | **P-value** |
| GO:0005615 | extracellular space | TF, OIH, ALB, IGLL1, P04210, BG | 0.0000008 |
| GO:0005576 | extracellular region | TF, OIH, ALB, IGLL1, P04210, P01875, BG | 0.000001 |
| GO:0044421 | extracellular region part | TF, OIH, ALB, IGLL1, P04210, BG | 0.00001 |
| GO:0046658 | anchored component of plasma membrane | TF | 0.007 |
| GO:0009986 | cell surface | TF, BG | 0.008 |
| GO:0031225 | anchored component of membrane | TF | 0.009 |
| GO:0009897 | external side of plasma membrane | BG | 0.008 |
| GO:0045121 | membrane raft | BG | 0.04 |
| GO:0098552 | side of membrane | BG | 0.05 |
| GO:0044459 | plasma membrane part | TF, BG | 0.06 |
| GO:0005794 | Golgi apparatus | BG | 0.06 |
|  | | | |
| **Go annotation of differentially expressed proteins in molecular function after 13-days of incubation compared to the control (6-days)** | | | |
| **Serial number** | **Annotation** | **Differentially expressed protein^1^** | **P-value** |
| GO:0045735 | nutrient reservoir activity | VTG2, VTG3 | 0.0000004 |
| GO:0005319 | lipid transporter activity | VTG1, VTG2, VTG3 | 0.000007 |
| GO:0003823 | antigen binding | P04210, P01875 | 0.0001 |
| GO:0070892 | lipoteichoic acid receptor activity | BG | 0.0003 |
| GO:0005041 | low-density lipoprotein receptor activity | BG | 0.001 |
| GO:0008035 | high-density lipoprotein particle binding | BG | 0.001 |
| GO:0019870 | potassium channel inhibitor activity | OIH | 0.001 |
| GO:0038187 | pattern recognition receptor activity | BG | 0.002 |
| GO:0008329 | signaling pattern recognition receptor activity | BG | 0.002 |
| GO:0030228 | lipoprotein particle receptor activity | BG | 0.003 |
| GO:0030169 | low-density lipoprotein particle binding | BG | 0.004 |
| GO:0071814 | protein-lipid complex binding | BG | 0.006 |
|  | | | |
| ^1^Differentially expressed protein. VTG1, VTG2, VTG3, OIH, IGLL1, P01875, ALB, and BG represent the gene names of vitellogenin-1 precursor, vitellogenin-2, vitellogenin-3, ovoinhibitor, immunoglobulin lambda light chain precursor, immunoglobulin Y heavy chain constant region, ovalbumin, and beta-2 glycoprotein-1 precursor respectively.List only the top 10-12 annotations for P values. | | | |
